# BacteSign: Building a Findable, Accessible, Interoperable, and Reusable (FAIR) Database for Universal Bacterial Identification

**DOI:** 10.3390/bios14040176

**Published:** 2024-04-05

**Authors:** Andre Childs, David Chand, Jorge Pereira, Swadeshmukul Santra, Swaminathan Rajaraman

**Affiliations:** 1 Department of Materials Science and Engineering, University of Central Florida, Orlando, FL 32816, USA; 2 NanoScience Technology Center, University of Central Florida, Orlando, FL 32826, USA; 3 Department of Mechanical and Aerospace Engineering, University of Central Florida, Orlando, FL 32816, USA; 4 Department of Chemistry, University of Central Florida, Orlando, FL 32816, USA; 5 Burnett School of Biomedical Sciences, University of Central Florida, Orlando, FL 32827, USA; 6 Department of Electrical and Computer Engineering, University of Central Florida, Orlando, FL 32816, USA

**Keywords:** impedance-based biosensor, antibiotic susceptibility assay, search query database, bacterial biofilms, Gram-negative bacteria

## Abstract

With the increasing incidence of diverse global bacterial outbreaks, it is important to build an immutable decentralized database that can capture regional changes in bacterial resistance with time. Herein, we investigate the use of a rapid 3D printed µbiochamber with a laser-ablated interdigitated electrode developed for biofilm analysis of *Pseudomonas aeruginosa*, *Acinetobacter baumannii* and *Bacillus subtilis* using electrochemical biological impedance spectroscopy (EBIS) across a 48 h spectrum, along with novel ladder-based minimum inhibitory concentration (MIC) stencil tests against oxytetracycline, kanamycin, penicillin G and streptomycin. Furthermore, in this investigation, a search query database has been built demonstrating the deterministic nature of the bacterial strains with real and imaginary impedance, phase, and capacitance, showing increased bacterial specification selectivity in the 9772.37 Hz range.

## 1. Introduction

Detection levels of pathogenic bacteria and other infectious diseases have become a major concern over the last few years due to the increase in multidrug-resistant (MDR) bacteria and events such as the recent global COVID-19 pandemic. Out of the 300 million bacterial infections a year, a staggering 4.95 to 16 million deaths are reported worldwide [1,2]. From reports, it is estimated that >250,000 of these infections will take place due to postoperative surgical sites [3,4,5,6]. These bacterial infections are caused by both Gram-negative and Gram-positive bacteria (GNB and GPB), with GNB-MDR being responsible for most intensive care unit submissions and morbidity [7,8]. These GNB pose significant risks to specifically immunocompromised patients undergoing, for instance, chemotherapy and organ transplantations (e.g., patients with liver cirrhosis). GNB were responsible for 59.6% of blood-stream infections in liver patients, where intravenous injection of the appropriate antibiotics is required to be administered within 24 h [9]. Thus, it is important to have a methodology to view any changes in relative real time that occur with bacterial strains that are high priority and common in hospitals, such as *Enterococcus faecium*, *Staphylococcus aureus*, *Klebsiella pneumoniae*, *Acinetobacter baumannii* (*A. baumannii*), *Pseudomonas aeruginosa* (*P. aeruginosa*), *Escherichia coli* also known as ESKAPE Bacteria, and their relatives such as *Bacillus subtilis* (*B. subtilis*) [10].

Bacteria exhibit two distinct growth modalities: a free-floating planktonic mode for rapid proliferation and a sessile biofilm mode [11]. The production of biofilms can be divided into five steps: (1) initial attachment, (2) monolayer growth, (3) multilayer growth, (4) maturation to extracellular matrix growth, and (5) 3-dimensional architecture and dispersion [12,13]. Once a biofilm infection is formed, it becomes difficult to treat due to the extracellular matrix produced by the developed bacterial microenvironments that evade immune response. In medical settings, biofilms can infect curtains, surgical equipment, catheters, artificial joints, and other implanted devices, becoming responsible for 65% of nosocomial infections, which may also result in the transition to one-time use of procedural equipment such as endoscopy equipment [14,15,16].

One of the best-known properties in the development of antibiotic resistance is that resistance is greater in biofilms as opposed to their planktonic cell state. Once these bacteria successfully infiltrate the body either through a wound or other means, they will begin delaying the body’s natural inflammatory healing process and form biofilms [17,18]. For instance moist tissue or injured mucous membranes are a well adapted environment for *A. baumannii*. Once exposed to the area of interest, infection begins first turning into a peau d’orange appearance eventually turning sandpaper-like with clear blood vessels indicating a severe infection [18]. *A. baumannii* is also able to weakly hydrolyze penicillin and carbapenem due to the possession of an intrinsic class D enzyme belonging to the OXA-1 group of enzymes [19]. A study performed by Kanno et al. found that *P. aeruginosa* develops biofilms within wounds in a short (for bacterial growth propagation) 8 h time frame [20]. A genetic basis for *P. aeruginosa* biofilm antibiotic resistance comes from the observation that surface-active rhamnolipid surfactants affect the architecture of biofilms, creating low pharmaceutical permeability [21]. *B. subtilis* biofilms have high population densities and form pellicles at air–liquid interfaces and, once mature form, organize spatiotemporal formations in long chains of nonmotile cells with an extracellular matrix of polysaccharides holding them together [22,23].

With the upward trend of region-specific antibiotic resistance and outbreaks in remote and densely populated areas around the globe, it is important to have a better understanding of the localized handling and the documentation of minimum inhibitory testing (MIC) of planktonic and biofilm-resistant strains. Similarly, it is paramount to publicize which antibiotics are effective against specific strains to help physicians design adequate patient treatments [24,25]. Unfortunately, current trends in data sharing are sparse and limited, as many databases are behind a private company paywall, providing revenue or localized to a specific region and/or hospital, which are rarely accessible to the public at large [26]. Imaging and genomic data of bacterial strains are densely populated in databases such as the International Nucleotide Sequence Database Collaboration (INSDC), MetaboLights, ProteomeExchange, and the BioImage Archive [27,28,29,30,31]. However, a large portion of microbial data can be filled with synthetic data created by companies such as Gretel.ai and Mostly.ai to provide training to machine learning models [32,33,34,35,36].

This use of synthetic data can also place constraints on principal component analysis, artificial intelligence, and machine learning models, which correspondingly translates into a large problem of enhanced morbidity, increased healthcare costs, reduced strategies for treatment, and overall public health analysis [31,37,38]. With the limited, accurate resources and synthetic information, it has recently been recommended that the data registered within these online ledgers needs to be (1) findable, (2) accessible, (3) interoperable, and (4) reusable, known as the FAIR Guiding Principles [39]. The development of novel biosensing tools that can monitor and provide accurate, time-sensitive data to the growing threat of antibiotic resistance among bacteria is desperately needed in order to reduce biased uncertainties and follow FAIR.

The current state-of-the-art biosensing technologies and conventional tests used to diagnose these bacterial infections include tools such as polymerase chain reaction (PCR), quartz crystal microbalance (QCM), surface plasmon resonance (SPR), surface-enhanced Raman scattering (SERS), fluorescence spectroscopy, and bacterial staining. The common theme with all of these techniques is that they take between 18 to 24 h to execute, are not species-specific, and require highly trained professionals in order to determine the nature and species of bacterial infection [40,41,42,43,44]. These issues necessitate the development of low-cost, easy-to-use, point-of-care biosensors as an extremely important topic for the community. Newer biosensing approaches, such as single-cell trapping and dielectrophoresis devices that utilize microfluidic chambers, have emerged and are usually time-consuming, complicated to design, can damage the cell walls, and only sense a few bacteria [45,46]. However, the number of bacteria is not deterministic to the diagnosis of bacteria affecting the patient or environment and needs to be increased as bacterial infections are often polymicrobial [16,47].

Nondestructive methods such as electrochemical bioimpedance spectroscopy (EBIS) have emerged as promising tools for real-time sensing in medical and industrial settings [48,49]. EBIS is an inexpensive technique that can become a useful tool to work alongside these state-of-the-art technologies in bioremediation spaces, where rapid detection and response are critical. EBIS can differentiate between GNB and GPB in their early stages before they become established and difficult to remove. It has also been shown to differentiate between biofilm-forming and nonbiofilm-forming bacteria [50]. Enabling EBIS on 3D printed devices allows for rapid design, affordability, and the possibility to print devices in different terrains ranging from Earth to space. Moreover, 3D printing has already been used for size separation [51], antibiotic resistance [52], and food microbiology biosensing [53]. The 3D printed surfaces provide irregularities arising from the processes themselves, which are suitable for biofilm formation along with enhanced signaling for bacterial analysis [48]. EBIS has already been used to study the effects of biofilm inhibition; however, there has been a lack of reports on EBIS and the use of selective media for bacterial sensing [54].

**Figure 1:**
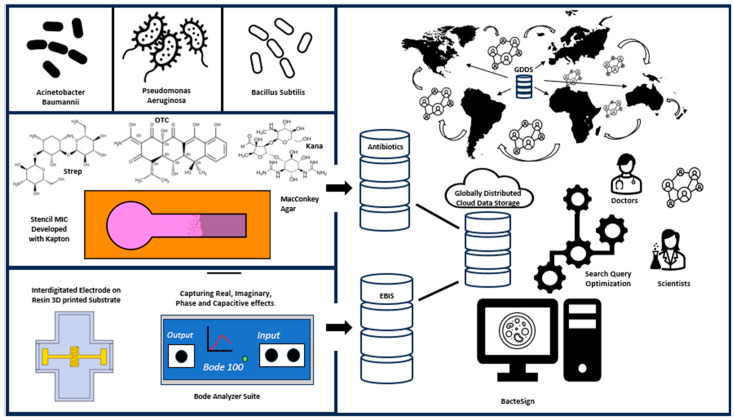
Depiction of the globally distributed immutable cloud database, with the inclusion of selective query searches for the optimized Kirby Bauer test and EBIS.

In this study, we chose MacConkey media (selective media) to investigate a noninvasive assay for detecting GNB biofilms without disrupting the bacterial growth pattern. With the use of selective media, clinicians can quickly distinguish the possibility of whether an organism is in GNB or GPB, increasing hospital safety and security. MacConkey agar only allows for GNB or opportunistic pathogens to grow on it due to the composition of bile salts, crystal violet, and lactose carbohydrate sources [55]. With the results from the EBIS and Kirby Bauer MIC stencil comparison test (extension from our prior work [56]), a search query database for determining which bacterial infection is present in clinical settings was developed (schematically depicted in Figure 1). As far as our knowledge goes, there are few papers that report the use of MacConkey media, and there are no databases that report the distinguishable features from electrochemical biological impedance spectroscopy or the increase in antibiotic dosage with minimum inhibitory concentration (MIC) tests.

## 2. Materials and Methods

### 2.1. Methods of Imaging

The following tools were used to image all the reported data in the Results and Supplementary Materials sections. The surface of the IDEs was analyzed using the Confocal Microscope (Keyence BZ-X800, Itasca, IL, USA).

### 2.2. Stencil Mask Fabrication for Minimum Inhibitory Concentration Tests

The optimized Kirby Bauer stencil mask for MIC tests was designed as described in Childs et al. [50]. Frozen bacteria culture stocks were stored in 20% (*v/v*) in Mueller-Hinton II Broth at −80° C in Thermo Scientific REVCO Freezer ULT2186-5AVA (Waltham, MA, USA). A culture of *P. aeruginosa* Schroeter Migula (ATCC 15692), *A. baumannii* Bouvet Grimont (ATCC 19606), and *B. subtilis Ehrenberg Cohn* (ATCC 11774) was cultured in MacConkey broth overnight at 150 rpm and 37 °C. Bacteria were inoculated onto the MacConkey Agar plates with the following OD_600_ equivalent of 1 × 10^8^ CFU, as shown in Table 1. Antibiotic disk diffusion assays were then performed in triplicates (i.e., *n* = 3) using oxytetracycline hydrochloride (7.5 ↔ 60 µg) (Alfa Aesar, Haverhill, MA, USA), streptomycin sulfate (2.5 ↔ 20 µg) (Fisher Bioreagents, Pittsburgh, PA, USA), kanamycin sulfate (7.5 ↔ 60 µg) (Fisher Bioreagents, Pittsburgh, PA, USA), and penicillin G (3 ↔ 24 µg) (Thermo Scientific, Waltham, MA, USA).

### 2.3. Laser Interdigitated Electrode (IDE) Substrate on µbiochamber Development

Laser micromachining patterning of the IDE design was performed in the direct write QuickLaze 50ST2 multimodal Neodymium-doped Yttrium Aluminum Garnet (Nd:YAG) laser (New Wave Research Inc., Fremont, CA, USA) using the green laser wavelength (525 nm) for ablation. The design dimensions created in SolidWorks (SolidWorks, Waltham, MA, USA) for the length (L), width (W), and spacing (S_p_) of the IDE were L = 2.5 mm, W = 560 µm, and S_p_ = 30 µm. The stencil mask for the IDE was aligned to the center of the µbiochamber using 300 HN Kapton masks produced in the Silhouette Cameo 4 plotter cutter using the parameters discussed in Childs et al. [56]. The metal deposition was performed in the Temescal E-beam evaporator (Ferrotec, Livermore CA, USA) with a chamber pressure of 1 × 10^−6^ torr. Ti (thickness of 50 nm) and Au (thickness of 150 nm) were deposited on the base of the µbiochamber using Ti, 4N5 purity pellets and Au, 5N purity pellets with deposition rates of 1.5 nm/s and 3.7 nm/s, respectively. The metal pellets were purchased from Kurt K. Lesker Company (Jefferson Hills, PA, USA). After, the deposition of the Ti/Au laser patterning of the IDE was performed in the QuickLaze 50ST2. The deposited IDE pad was designed to be 2.5 mm in width and 6 mm in length, with the trace to the left and right being 1 mm and wired pads being 5 mm and 1.75 mm in length.

### 2.4. Bacterial Interdigitated Electrode µbiochamber Assembly

**Figure 2:**
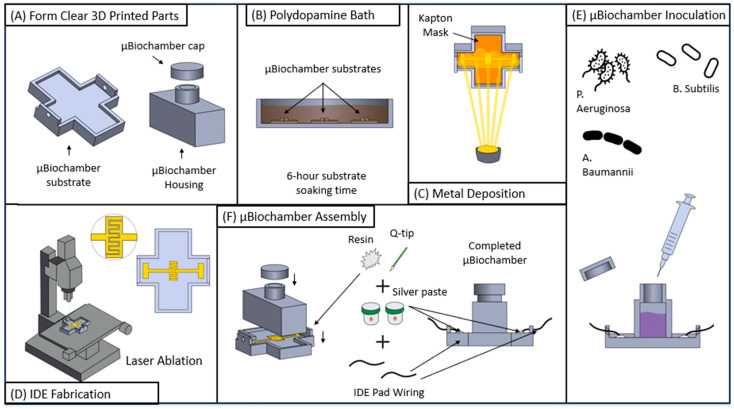
Process flow for the µbiochamber. (**A**) Three-dimensional printed substrate, cap, and inoculation chamber. (**B**) Six-hour polydopamine bath. (**C**) stencil mask and Ti/Au deposition. (**D**) Laser ablation to fabricate the IDE (**E**) µbiochamber chamber assembly. (**F**) Inoculation of various bacteria.

The IDE µbiochamber substrate was prepared using an Asiga MAX X27 385 nm printer (Asiga Ltd., Alexandria, Australia) using FormLabs clear resin 4 (FormLabs, Boston, MA, USA). The µbiochamber housing and cap were printed in the FormLabs Form 3 µSLA printer using FormLabs clear resin V4. The µbiochamber was developed in two parts: (1) the µbiochamber housing and cap for containing the broth and the bacterial colonies and (2) the µbiochamber substrate base plate where the IDE resided. The µbiochamber size was 25.4 mm in length and 9.5 mm in width with 9.5 mm in height, allowing for a volume of 2.29 mL of the cultured bacteria. The base plate of the two-part µbiochamber was fabricated for the chamber to fit seamlessly inside. The µbiochamber was assembled, as shown in Figure 2A–D. Figure 2A shows the 3D µbiochamber and cap, which were printed on the Form 3 printer using clear resin, while the substrate of the device was printed on the Asiga 3D printer to reduce surface roughness [57,58]. In Figure 2B, once printed, the µbiochamber substrates were placed in a polydopamine (PDA) solution (1:1 M Dopamine Hydrochloride (Thermo Scientific, Waltham, MA, USA) to 1-ethyl-3-(3-dimethylamine propyl) carbodiimide HCl (Thermo Scientific, Waltham, MA, USA) and soaked for 6 h to improve gold adhesion [58,59]. After 6 h, the µbiochamber substrates were fitted with the Kapton mask and subject to metal deposition, as discussed in Section 2.3. Following deposition, for sterilization, the µbiochamber cap, µbiochamber housing, and µbiochamber substrate were placed in a 70% ethanol solution for sterilization in the biosafety cabinet and left to air dry. Subsequent to drying, uncured resin was placed on the bottom edges of the housing chamber and attached to the IDE substrate; the cap is sequentially attached to the housing chamber for a tight seal to prevent aeration during culturing experiments. The assembled device was subsequently placed in a FormLabs UV-cure, located inside a biosafety cabinet for 5 min to negate biocontamination. Once cured, silver paste was used to connect the left and right IDE pads with 2.5-inch-long wires (VT Corporation, B-30-1000, Bengaluru, India). The wires were inserted through the holes of the IDE substrate and attached with the silver paste (AI Technology, Princeton, NJ, USA Parts A&B EG8020). The devices were then left to cure overnight in a Thermo Fisher Precision Oven (Model: PR305225M, Thermo Fisher, Waltham, MA, USA) for 24 h at 60 °C. Once completed, the uncured resin was used to cover the electrical wire connection and UV-cured for 5 min to secure the connection.

### 2.5. Bacteria Strain Handling, Growth Conditions, and Media Culture for EBIS

Frozen bacteria culture stocks were stored in 20% (*v/v*) in Mueller-Hinton II Broth at −80 °C in Thermo Scientific REVCO Freezer ULT2186-5AVA (Waltham, MA, USA). *P. aeruginosa* Schroeter Migula (ATCC 15692), *A. baumannii Bouvet Grimont* (ATCC 19606), and *B. subtilis* Ehrenberg Cohn (ATCC 11774) were cultured in MacConkey broth overnight at 150 rpm and 37 °C. The bacterial broth was then diluted and inoculated into the µbiochamber with the following OD_600_ equivalent of 1 × 10^8^ CFU, as shown in Table 1, and then sealed to prevent aeration. The µbiochamber was subsequently placed in the incubator for 48 h and recorded for impedance measurements (*n* = 3), as shown in Figure 2E.

### 2.6. Impedance Measurements

The µbiochamber with the various bacteria listed *P. aeruginosa*, *B. subtilis*, and *A. baumannii*, along with MacConkey broth control devices, were removed from the incubator and immediately subjected to impedance measurements for repeatability. The experiments were performed in triplicates using three new microfabricated devices (*n* = 3). The full range of frequency spectra was obtained over the range of 1 Hz–10 MHz using alternating current with the Vector Network Analyzer Bode 100 from Omicron Labs (Vorarlberg, Austria). Complex impedance, phase, and capacitance measurements were obtained at the intervals of 0, 1, 2, 4, 6, 8, 12, 18, 24, 36, and 48 h for bacterial growth and nonbacterial (control) growth. The control IDE and Bacterial growth on the IDE can be seen in Supplementary Figures S2–S7.

### 2.7. Database Building

A Caspio Database (Caspio, Sunnyvale, CA, USA) was built with Caspio.com, a graphical programming interface. The MIC results from the Kirby Bauer comparison and stencil susceptibility tests with the results from oxytetracycline hydrochloride (7.5 ↔ 60 µg), streptomycin sulfate (2.5 ↔ 20 µg), kanamycin sulfate (7.5 ↔ 60 µg), penicillin G (3 ↔ 24 µg), and the full spectrum IDE data (control and bacterial growth) were placed into the database and queried using IF, OR, and AND logic statements, against real impedance, imaginary impedance, phase, and capacitance in the intervals of 0, 1, 2, 4, 6, 8, 12, 18, 24, 36, and 48 h. The results were also queried against the MIC stencil antibiotics and the corresponding pharmaceutical dosages that were used for the comparative Kirby Bauer study. These measurements were input and queried using the built Caspio database to present the results of the query. Full 48 h metadata spectrums for all bacteria tested with their standard deviation as well as searching the query are included in Supplementary Materials—Tables S1–S5 and Figures S8–S12.

## 3. Results

### 3.1. Optimized Kirby Bauer Stencil Mask

**Figure 3:**
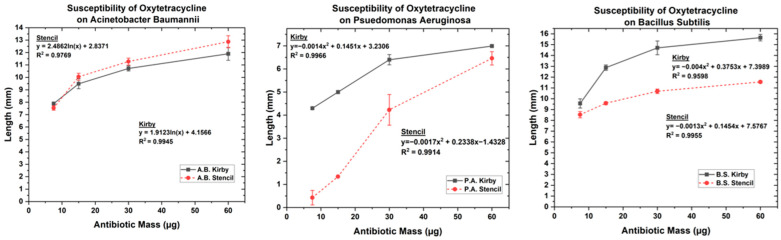
Antibiotic susceptibility test of oxytetracycline on (**Left**) *A. baumannii*, (**Center**) *P. aeruginosa*, and (**Right**) *B. subtilis*.

**Figure 4:**
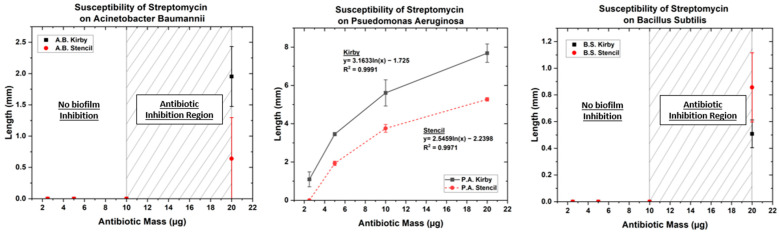
Antibiotic susceptibility test of kanamycin on (**Left**) *A. baumannii*, (**Center**) *P. aeruginosa*, and (**Right**) *B. subtilis*.

**Figure 5:**
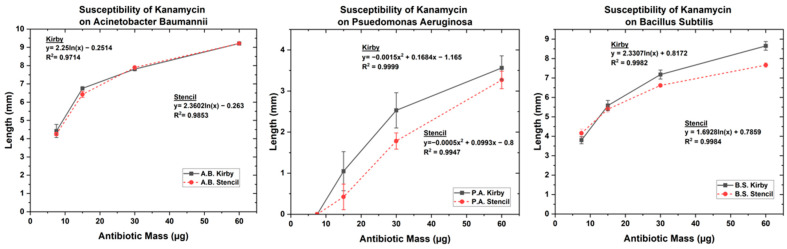
Antibiotic susceptibility test of streptomycin on (**Left**) *A. baumannii*, (**Center**) *P. aeruginosa*, and (**Right**) *B. subtilis*.

The results of the MIC are shown in Table 2. Our findings show that the radius and stencil area of inhibition for the antibiotics both changed with the different bacteria, as shown in Figure 3, Figure 4 and Figure 5. The MIC showed high resistance to streptomycin and penicillin G; however, *P. aeruginosa* was susceptible to streptomycin, and *B. subtilis* was susceptible to penicillin G. The MIC results showed that *A. baumannii* and *B. subtilis* possessed high susceptibility for both kanamycin and oxytetracycline, while *P. aeruginosa* showed intermediate susceptibility. The governing equations regarding the growth inhibition for *P. aeruginosa*, *A. baumannii*, and *B. subtilis* in the presence of oxytetracycline, kanamycin, streptomycin, and penicillin G follow one of the two equations below.

Here, y is the radius or length of inhibition obtained from the traditional Kirby Bauer and modified microstencils. Additionally, X represents the mass of antibiotics inoculated in the paper disk used for the test.

Natural Log:

y = C_1_ln(X) + B (1)

Polynomial:

y = C_1_X+ C_2_X^2^ + B (2)

When oxytetracycline and kanamycin were tested for susceptibility against *A. baumannii*, the inhibition pattern followed the form of equation 1, whereas *A. baumannii’s* resistance to streptomycin and penicillin G was only overcome at a concentration double the starting concentration of 10 µg and 6 µg, respectively, corresponding to a low susceptibility. On the other hand, *P. aeruginosa* showed a distinct difference in response to the pharmaceuticals following the polynomial (Equation (2)) for both oxytetracycline and kanamycin while following a natural logarithmic (Equation (1)) for streptomycin. When penicillin G was tested against *P. aeruginosa*, there was no growth inhibition. For the effect of oxytetracycline on *B. subtilis*, a second-order polynomial is followed, whereas the susceptibility for kanamycin followed a natural logarithmic equation. When *B. subtilis* was tested for its resistance to streptomycin and penicillin G, no growth inhibition occurred until 20 µg and 24 µg were tested, respectively, as shown in Supplementary Materials—Figure S1. Overall, these results show that nonsusceptible bacteria showcase an immediate response when the antibiotic is able to overcome its resistance due to increasing dosage, whereas susceptible bacteria follow logarithmic or second-order polynomial trends similar to other reports in the literature [56,60,61]. Indicative enough, these equations can be followed for direct correlations when studying MDR and antimicrobial pharmacokinetic properties.

### 3.2. Electrochemical Biological Impedance Spectroscopy (EBIS)

#### Characterization of Interdigitated Electrode

**Figure 6:**
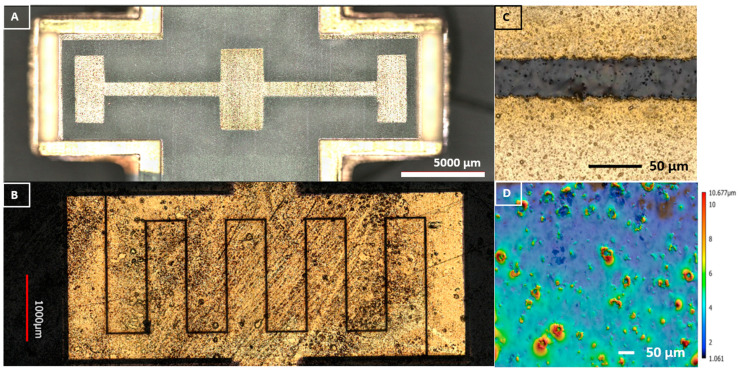
(**A**) Image of the metal deposition after stencil mask removal. (**B**) Center pad after laser IDE ablation. (**C**) Laser confocal image of IDE gap. (**D**) Laser confocal height map of surface roughness of resin substrate.

Using the power of laser ablation for the micromachining approach allows for on-the-spot prototyping as designs can be changed extremely quickly, based on needs, skipping the requirement for developing new molds or photolithographic masks [56,62]. Figure 6A–C depict confocal images of the IDEs. The final stencil mask designs measured an average *(n = 3)* central pad width of 2514.12 µm, resulting in a 0.56%percent error, and an average central pad length of 5983.41 µm, representing a 0.27% percent error. The left and right pad traces measured 977.37 µm and 1025.35 µm with percent errors of 2.29% and 2.5%, respectively. The left pad width (LPW) measured 1669.906 µm, and the left pad length (LPL) was 5023.85 µm, resulting in 1.78% and 0.47% percent errors from design dimensions, respectively. The right pad width (RPW) and right pad length (RPL) measured 1752.50 µm and 5059.74 µm, resulting in 3.04% and 1.18% percent error, respectively. The final finger width and spacing of the IDE measured 561.59 µm and 33.03 µm, respectively, resulting in a ratio 17:1. The surface roughness of the resin substrate printed in the Asiga with FormLabs clear resin 4, when measured with confocal laser microscopy had a surface roughness with an arithmetical mean of 0.746 µm while the sum of the largest pit to largest peak height was 9.81 µm for surfaces covering an average area shown in Figure 6D. This surface roughness is of important significance as it has been shown that a rough surface can provide an increase in signal for bioimpedance spectroscopy [63].

**Figure 7:**
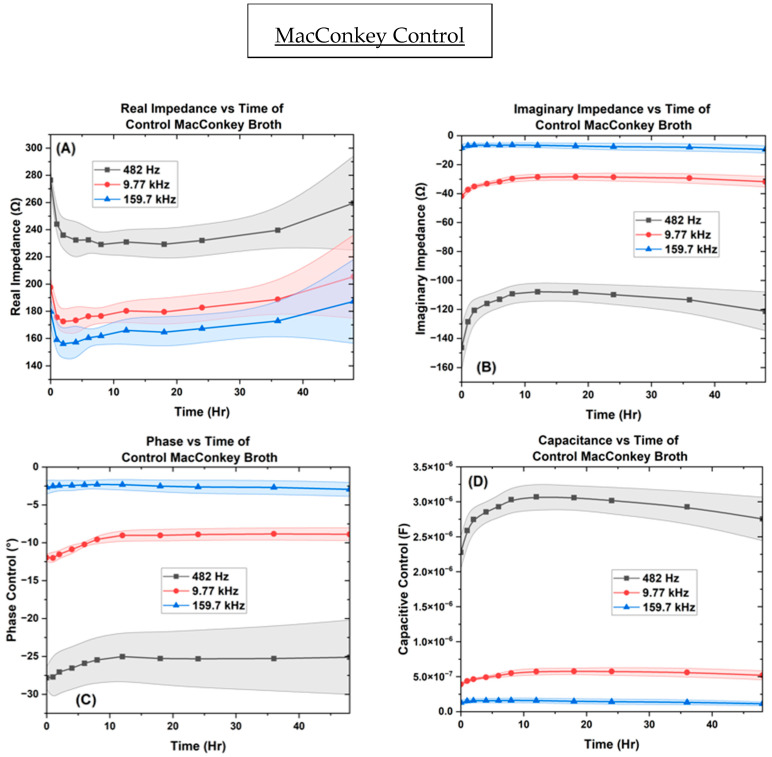
The control of MacConkey broth on the interdigitated electrode depicting the ((**A**) real impedance vs. time, (**B**) imaginary impedance vs. Time, (**C**) phase vs. time, and (**D**)) capacitance vs. time.

Throughout the impedance measurements, as the alternating current passes through the MacConkey media containing the microbial growth, the metabolic activities become electrically detectable due to the nutrients and ionic efflux components from bacteria in the media [53,64]. Below in Figure 7, we can observe that the control provided stable measurements in real impedance, imaginary impedance, phase, and capacitance in the frequency points of 482.31 Hz, 9.7 kHz, and 159.7 kHz, respectively. The real impedance provides results that are stable between [229 ↔ 276 Ω], [172 ↔ 205 Ω], [156 ↔ 187 Ω] across the 48 h. For the imaginary impedance, the control remained stable [−107 ↔ −146 Ω], [−28 ↔ −41 Ω], and [−6 ↔ −9 Ω] across the same frequencies. The phase results were also stable between [−25 ↔ −27°], [−6 ↔ −9°], and [−8 ↔ −12°], reaching equilibrium in 8 h at the same frequency points, respectively. For the capacitance, the recurring measurements remained between [2.27 ↔ 3.07 µF], [0.39 ↔ 0.52 µF], and [0.11 ↔ 0.16 µF], reaching equilibrium in 8 h. Figure S2 shows the surface of the control IDE in the SEM after 18 and 48 h.

**Figure 8:**
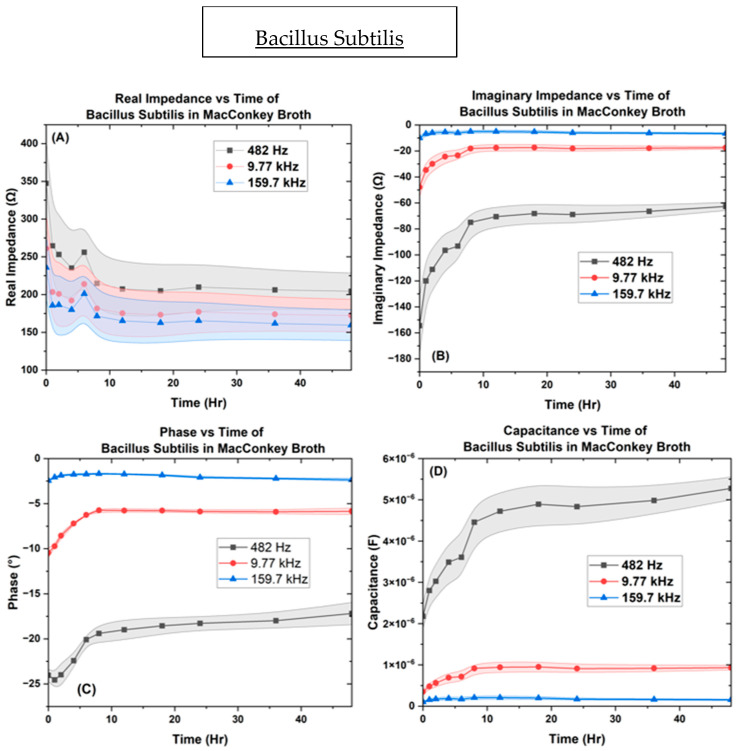
The µBiochamber inoculated with *B. subtilis* on the IDE depicting the (**A**) real impedance vs. time, (**B**) imaginary impedance vs. time, (**C**) phase vs. time, and (**D**) capacitance vs. time.

In Figure 8, results from the various impedance measures for *B. subtilis* when it is inoculated into the µbiochamber at a concentration of OD_600_ = 1.0 for 48 h at frequency points of 482.31 Hz, 9.7 kHz, and 159.7 kHz. The real impedance varied between [204 ↔ 348 Ω], [170 ↔ 261 Ω], and [159 ↔ 235 Ω] across the 48 h time frame. The values for the imaginary impedance were [−62 ↔ −154 Ω], [−17 ↔ −48 Ω], and [−5 ↔ −10 Ω]. The phase values measured between [−17 ↔ −25°], [−5 ↔ −11°], and [−1.6 ↔ −2.51°], reaching equilibrium in 8 h, while the capacitance measured between [2.17 ↔ 5.27 µF], [0.34 ↔ 0.94 µF], and [0.1 ↔ 0.21 µF], reaching equilibrium in 8 h. Figure S3 shows B. subtilis biofilm growth over time.

**Figure 9:**
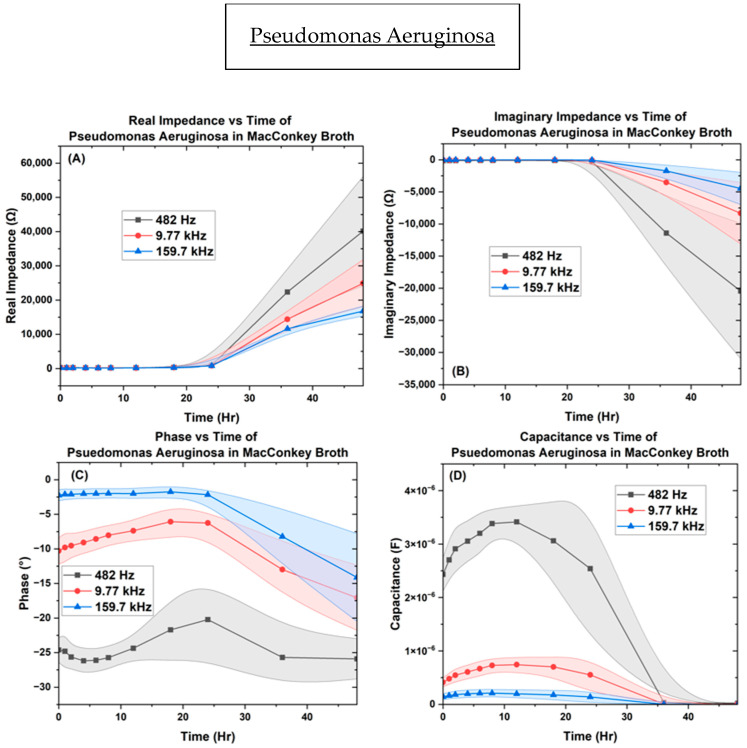
The µBiochamber inoculated with *P. aeruginosa* on the interdigitated electrode depicting the (**A**) real impedance vs. time, (**B**) imaginary impedance vs. time, (**C**) phase vs. time, and (**D**) capacitance vs. time.

In Figure 9, results from *P. aeruginosa* inoculated into the µbiochamber at a concentration of OD_600_ = 0.4 for 48 h at the frequency points of 482 Hz, 9.7 kHz, and 159.7 kHz are showcased. The real impedance varies from [200 ↔ 40,140 Ω], [164 ↔ 24,920 Ω], and [153 ↔ 16,800 Ω] with higher fluctuations at 48 h. The imaginary impedance, shown in Figure 9, top right, allowed for *P. aeruginosa* to remain bound between the conditions of [−97 ↔ −20,500 Ω], [−22 ↔ −8350 Ω], and [−5 ↔ −4500 Ω] with higher fluctuations between samples at 48 h. For the phase, the measured values were between [−20 ↔ −26.2°], [−6 ↔ −17°], and [−1.4 ↔ −14°], showing a resistive behavior at 12 h and then slowly decreased after 12 h, indicating capacitive behavior in the region. The capacitance measurements remained between [2.0 ↔ 0.02 µF], [0.41 ↔ 0.002 µF], and [0.14 ↔ 0.0002 µF], showing a sharp decrease in capacitance after 12 h. Figures S4−S6 show the biofilm growth of *P. aeruginosa* over time.

**Figure 10:**
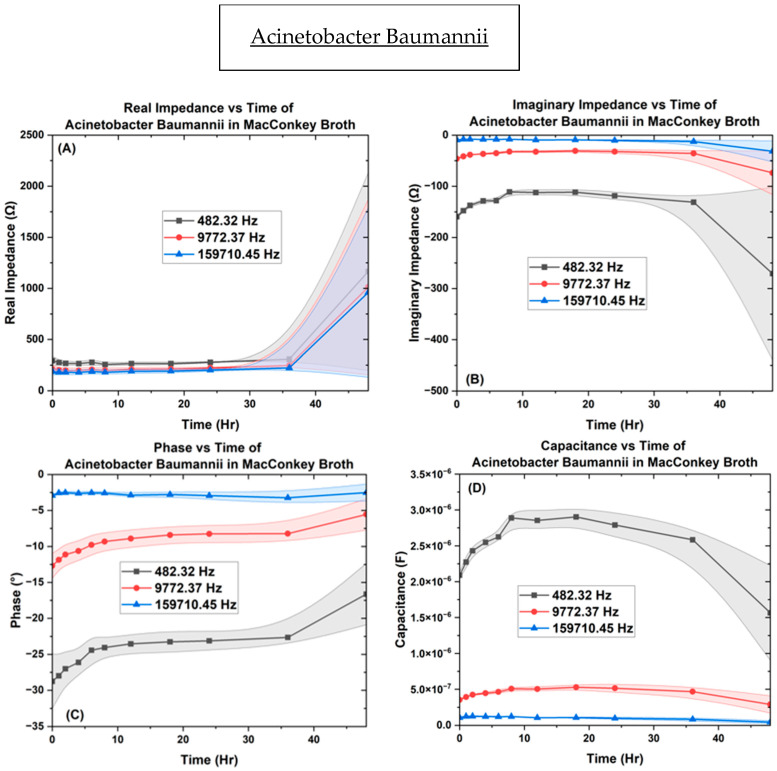
The µBiochamber inoculated with *A. baumannii* on the interdigitated electrode depicting the (**A**) real impedance vs. time, (**B**) imaginary impedance vs. time, (**C**) phase vs. time, and (**D**) capacitance vs. time.

In Figure 10, results from *A. baumannii* inoculated into the µbiochamber at a concentration of OD_600_ = 0.1 for 48 h at the same measured frequencies are detailed. The real impedance measured between [250 ↔ 1165 Ω], [195 ↔ 1013 Ω], and [180 ↔ 1000 Ω] with higher fluctuations at the 48 h mark but not as high as *P. aeruginosa*. In Figure 10, for imaginarympedance, the *A. baumannii* measured [−110 ↔ −271 Ω], [−30 ↔ −74 Ω], and [−8 ↔ −32 Ω], respectively. The phase measured [−16 ↔ −29°], [−5 ↔ −13°], and [−2.5 ↔ −3.3°], then at 36 h showing a rise in the resistive components in the same frequencies. The capacitance measured between [2.3 ↔ 0.02 µF], [0.39 ↔ 0.003 µF], and [0.139 ↔ 0.0003 µF], with a rise until 18 h, then a gradual decrease until 36 h, along with a sharp decrease after 36 h. Figure S7 shows the biofilm growth of *A. baumannii* over time. 

**Figure 11:**
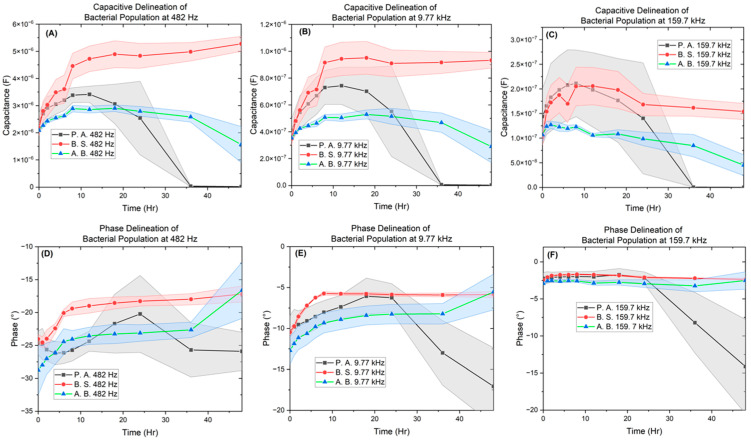
Image of capacitive (**Top**) and phase (**Bottom**) bacterial delineation plots. (**A**) Capacitance and (**B**) phase at 482 Hz. (**B**) Capacitance and (**E**) phase at 9.77 kHz. (**C**) capacitance and (**F**) phase at 159.7 kHz. The lower impedance frequencies 482 and 9.77 kHz show more separation than 159.7 kHz.

**Figure 12:**
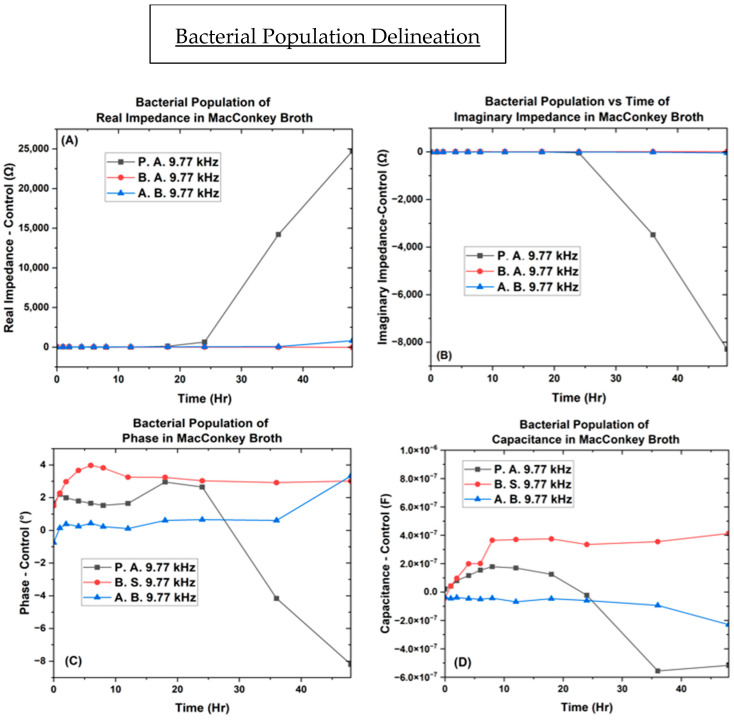
Bacterial population separation plot. Bacteria in MacConkey broth on the interdigitated electrode depicting the (**A**) real impedance vs. time, (**B**) imaginary impedance vs. time, (**C**) phase vs. time, and (**D**) capacitance vs. time.

Figure 7, Figure 8, Figure 9 and Figure 10 depict the change occurring within the IDE vs. time at the three selected frequencies of 482.32 Hz, 9772.37 Hz, and 159,710 Hz for the *B. subtilis*, *P. aeruginosa*, and *A. baumannii* strains of bacteria. In Figure 11, the separation of bacteria is shown with a high standard deviation shown in supplementary belonging to *P. aeruginosa*, which could be due to *P. aeruginosa’s* ability to have large genetic diversity, phenotype plasticity, and heterogeneity in clinical isolates [65]. It was also shown that after the 11th hour, *P. aeruginosa* reached a high experimental value of 10^15^ Ohms, by Chabowski et al. [66]. When this data was subtracted by the MacConkey broth control (Figure 12), it is observed in the capacitive analysis, the lag adaption phase occurs between 0 ↔ 2 h and the log phase between 2 ↔ 6 h, which is characterized by the sharp rise of *P. aeruginosa* and *B. subtilis*. The stationary phase occurs between 8 ↔ 12 h for *P. aeruginosa* following bacterial growth curves, and *B. subtilis* shows a stationary phase from 8 ↔ 36 h. The capacitive effect of *A. baumannii* is not indicative of a substantial increase in bacterial growth; however, the sharp rise is observed in the phase graph at 36 h, indicating that *A. baumannii*’s stationary phase changes at 36 h as indicated in the rise in real impedance and phase along with the gradual decrease in capacitance [53,67].

This change is also indicated to begin for *P. aeruginosa* at the 18 h mark, as shown by the rise in real impedance, decrease in imaginary impedance, and sharp decrease in capacitance. These quantitative characteristics allow for the determination of unknown samples as the ranges for the various bacteria are delineated from one another at the separate single frequency point values, with the widest range occurring at 9.77 kHz.

Thus, the data from this focused study indicates that the values for the three bacteria and control are vastly different across the 48 h culturing time frame. *P. aeruginosa* shows the greatest rise in real impedance and changes in phase, while *A. baumannii*’s trend remains stable until 36 h across real, imaginary phase, and capacitance. *B. subtilis* produces the largest capacitive effect of the three reaching along with its unique point of interest at the 6 h point, marking the transition from the log to stationary phase.

These distinguishable parameters of real and imaginary impedance, phase, and capacitance showcase the bacteria’s metabolic lag phase, exponential log phase, and stationary transition.

## 4. Discussion

The tested antibiotics on bacteria all either showed natural logarithmic, second-order polynomial equations or a sudden increase in bacterial biofilm inhibition. The function of the equation states that when following a natural logarithmic, the Δy (length) will increase slowly as the antibiotic (X) mass increases, with an eventually decreasing Δy as it gets to a saturation point. The second-order polynomial functions coming from the susceptibility tests show minuscule a(C_1_) and b(C_2_) constants, implying that there is a relatively slow increase in inhibition growth. The second order polynomial function would insinuate that bacteria are more susceptible to the pharmaceuticals than bacteria adhering to the natural logarithmic equations. The bacteria that are able to prevent the pharmaceuticals from disrupting their membrane until there is a sudden change in the area of inhibition “defend” themselves from cellular lysis by having enough ions due to efflux in their membranes and can divide at rates quick enough to maintain these ions.

Oxytetracycline, being lipophilic, inhibits protein synthesis in bacteria by diffusing through the porin channels and binding to the 30S ribosomal subunit caused by the hydroxyl groups located at C10 and C12. It binds to the 30S ribosomal subunit and prevents the aminoacyl-tRNA from binding to the A site of the ribosome [68,69,70]. Kanamycin, an aminoglycoside favoring polar and anionic phosphate head groups, is able to penetrate through the permeable bacterial cell membranes through passive diffusion and target RNA to inhibit replication [71,72]. The reason for the antibiotic defiance to streptomycin and penicillin G could be due to the diffusion of the therapeutics inside the biofilm being affected by the hydrophobicity of bacterial cell walls. The extracellular polymeric substances such as pili are also responsible for the low penetration of the antibiotic [73]. In addition to the exopolymeric substances, such as glycol proteins, pili, and peptidoglycan, that prevent pharmaceuticals from causing cell lysis in bacteria [74], outer membrane proteins, being the most abundant surface proteins on the pathogens, are involved in increased resistance and the formation of stable biofilms. Members of *A. baumannii* can contribute to the virulence potential as members of the outer membrane proteins have been determined to contribute significantly to the disease-causing potential of the pathogen [75]. Overall, it is the outside membrane that delegates the permittivity of antibiotics; the glucose polymers may prevent antibiotics of antibiotics: the exomoers are first inline as a defense preventing antibiotics from reaching their binding sites. [17,76].

The greater the capacitance, the more energy is stored in the system. Here, we see that *P. aeruginosa* begins to show a decrease in capacitance earlier than *A. baumannii*, whose phase begins to increase at 36 h, along with a decrease in capacitance at 36 h. This observed change shown by the real impedance cannot be conclusively attributed to cell death but is indicative of a physiological change, as live cells are known to increase capacitance and conductivity with time [53,67]. When the phase angle approaches 0° or 90°, it is primarily caused by resistance or influenced by reactance respectively. even though these pathogens each possess significant differences in their metabolism, contributing to their vastly different impedance and capacitance spectra. For example, *A. baumannii* is nonmotile as compared to *P. aeruginosa* and *B. subtilis*, which possess flagellates and are mobile. Motility allows bacteria to translocate themselves into microenvironments that are conducive to growth. When bacteria are suspended in nutrient broth, their growth is dependent on the concentration of oxygen. Obligated aerobes, such as *A. baumannii*, proliferate close to the surface of the liquid, where the concentration of oxygen is greatest. On the other hand, *P. aeruginosa* and *B. subtilis* are facultative anaerobes, which enables them to multiply throughout the broth at different rates [9,77,78,79]. In turn, the microenvironment in which bacteria prefer to grow affects the cell density near the electrodes of the µbiochamber.

For microbiological metabolism monitoring, it is found that when capacitance increases, the real impedance decreases [53,67]. The reason this occurs is due to the ionic compounds associated with the bacterial polysaccharides’ outer membranes, which would increase the conductance of the electrode. Contrary to what papers have stated showing an increase in impedance from cell density due to lipids, our results show that it is only upon the change in the stationary phase of the bacteria cells that real impedance begins to rise, which could be due to the size of the electrode or choice of media [80]. The ionic distributions dictate cell density or conductance, indicating that the cells did not impede the movement of ions in the medium or around the electrode. Along with the polysaccharides, the cytoplasmic membranes enhance conductivity in media [81]. Thus, microbial growth and metabolism have been shown to have a better resemblance through capacitive measurements rather than conductive data. It has also been shown that capacitive effects are less prone to fluctuations as they arise from polarization at the electrode–liquid interface [82,83,84,85].

Despite the resultant bacterial separation plot in Figure 11, further studies need to be performed on the antagonistic properties of specific bacterial functions. The constant phase and capacitance shown by *B. subtilis* are unique, showing a stagnation in all parameters studied parameters after after 8 h. It has been shown upon ligand binding, *B. subtilis* releases a large depot of pyridine-2,6-dicarboxylic acid a large peptidoglycan structure along with its predominate calcium cations, that degrade the spore cortex and prevent it from expanding. [86,87,88,89,90]. Recently gaining attention are the early sporulating Spo0A-active cells as an alternative to antibiotics due to a mechanism known as the cannibalism of siblings. The early cells cause the lysis of the dormant Spo0A sister cells by exporting an antibiotic-like sporulation killing factor (SKF) to which they are immune. [91]. *P. aeruginosa’s* fluctuation could be due to it‘s release of pyocyanin a molecule that goes undergoes redox reactions and causes it’s planktonic green coloration [92]. With this consideration, it would be interesting to add additional studies containing different optical densities of *B. subtilis, P. aeruginosa* and *A. baumannii* for comparisons. The use of different OD’s at hand will also be beneficial for the database, the reason being that if unknown contaminants are present in hospitals or other environments, there will be no deterministic optical density or CFU to begin the inoculation and impedance studies; only a binary hypothesis based upon standard preliminary testing such as the high-throughput MIC. The development of a method where unknown pathogens can be studied is the key to beginning this analysis.

Though the imaginary impedance has not been explored to our knowledge, we believe there is more to be gained by studying all bacterial growth kinetics. As shown in our findings in Figure 9 and Figure 10, the imaginary impedance spectra are different for *P. aeruginosa*, *B. subtilis*, and *A. baumannii*. Even though it has not been reported in the literature, we believe that the imaginary impedance is worth exploring, the reason being once the metadata of the database has increased, it will take more than a selective capacitive measurement to determine which unknown bacteria is present within a patient or scientific study as it has been shown that bacterial populations will also be normally distributed [93].

The data indicates that real and imaginary impedance show little variation in measurements until after the 36 h mark, which may be useful to improve the sensitivity of the device. The collection of bacterial isolates in the database, taken over the interval of time, was shown as searchable and repeatable in the regions of phase and capacitance, first becoming distinguishable at the 2 and 8 h mark, respectively.

## 5. Conclusions

In this work, we have presented the capability of using localized bacterial microenvironments for antibiotic susceptibility assays that are comparable with the Kirby Bauer disc diffusion method. The MICs showed an increased inhibition size with increased dose efficiency equivalating to logarithmic and polynomial equations when testing kanamycin, oxytetracycline, penicillin G, and streptomycin. We have also presented the development of a distinct stencil mask technology for IDE characterization using laser ablation for enhanced resolution on 3D printed substrates. Using this IDE, we recorded four parameters of bacterial biofilm growth, indicating that EBIS can be used to determine bacterial species at the 9.77 kHz frequency point at first the 2 and 8 h time points. A Caspio database was developed with data collected from two key assays: the MIC and EBIS assays. The variations in the low-cost, highly sensitive microbiological assays proved to be searchable in the query-enabled database, demonstrating that 3D printed substrates have the potential to become widely used tools in bacterial exploration, being of huge value extending to pharmaceuticals, biotechnology, food industries, and microbial conservation.

## Figures and Tables

**Table 1:** OD_600_ of bacteria.

Bacteria	OD_600_
*A. baumannii*	0.1
*P. aeruginosa*	0.4
*B. subtilis*	1.0

**Table 2:** Kirby Bauer and optimized stencil MIC comparison.

Antibiotic	Antibiotic Mass (µg)	A.B Kirby (mm)	A. B. Stencil (mm)	B. S. Kirby (mm)	B.S Stencil (mm)	P.A. Kirby (mm)	P.A Stencil (mm)
Oxytetracycline	7.5	7.89	7.53	9.56	8.52	4.29	0.42
	15	9.485	10.05	12.87	9.58	4.99	1.34
	30	10.72	11.27	14.71	10.69	6.39	4.23
	60	11.89	12.87	15.64	11.55	6.99	6.46
Kanamycin	7.5	4.42	4.25	3.8	4.15	0	0
	15	6.75	6.42	5.57	5.39	1.048	0.42
	30	7.81	7.89	7.17	6.61	2.53	1.78
	60	9.21	9.21	8.65	7.66	3.56	3.27
Streptomycin	2.5	0	0	0	0	1.09	0
	5	0	0	0	0	3.45	1.93
	10	0	0	0	0	5.61	3.75
	20	1.95	0.64	0.51	0.86	7.68	5.27
Penicillin G.	3	0	0	0	0	0	0
	6	0	0	0	0	0	0
	12	0	0	0	0	0	0
	24	0	0	5.13 *	0.36	0	0

* Not subtracted as stated in [50]. A.B. = *A. baumannii*, B.S. = *B. subtilis*, and P.A. = *P. aeruginosa* (calculated with *n* = 3 samples).

## Data Availability

All database data have been published within the supplementary.

## Supplementary Materials

The following supporting information can be downloaded at: https://www.mdpi.com/article/10.3390/bios14040176/s1, Table S1: Kirby Bauer and optimized stencil MIC comparison, Figure S1: Antibiotic susceptibility test of penicillin G. on *Bacillus subtilis*; Table S2: Real impedance at frequencies 482 Hz, 9.77 kHz, and 159.7 kHz for the Control, *A. baumannii, P. aeruginosa, and B. subtilis*. Table S3: Imaginary Impedance at frequencies 482.32 Hz, 9.77 kHz, and 159.7 kHz for the Control, *A. baumannii, P. aeruginosa, and B. subtilis*. Table S4: Phase at frequencies 482 Hz, 9.77 kHz, and 159.7 kHz for the Control, *A. baumannii, P. aeruginosa, and B. subtilis*. Table S5: Capacitance at frequencies 482 Hz, 9.77 kHz, and 159.7 kHz for the Control, *A. baumannii, P. aeruginosa, and B. subtilis*. Figure S2: (A) SEM image of the control IDE after 18 h. (B) Confocal Height map of the control IDE after 48-h, showing no change on the surface of the IDE or in the spacing. Figure S3 (A) SEM image of *B. subtilis* 12-h bacterial growth. (B) Confocal Height map of *B. subtilis* 24-h, showing cell growth in IDE gap. Figures S4: (A) Laser Confocal Image showing biofilm growth in the IDE gap. (B) SEM picture showing IDE surface covered with biofilm. Figure S5. (A) SEM close up of *Pseudomonas aeruginosa* dried biofilm growth. (B) Close up of dried biofilm growth. Figure S6. SEM close up biofilm growth showing individual cells forming biofilm for *Pseudomonas aeruginosa*. Figure S7: (A) Confocal Height Image of the interdigitated electrode gap with *A. baumannii* cell chains forming. (B) SEM image showing *A. baumannii* biofilm cells at the 24-h point. Figure S8: Interdigitated electrode partitioned database drop down and enterable standard deviation search menu. Figure S9. Using the dropdown menu for the Interdigitated electrode sharded database drop down menu. Figure S10. Not using the dropdown menu and including all hours leads to a multitude of options for bacterial discovery. Figure S11: Entering an Antibiotic Mass and limiting the search with a lower and upper bound Susceptibility test values. Figure S12: Using lower and upper bounds as a manual entrance for standard deviation
