# Supplementary material for: BacteSign: Building a Findable, Accessible, Interoperable, and Reusable (FAIR) Database for Universal Bacterial Identification
Source: Biosensors (Basel). 2024 Apr 5;14(4):176. doi: 10.3390/bios14040176 (PMC11047924; doi:10.3390/bios14040176)
Supplement: Supplementary file 1 [file biosensors-14-00176-s001.zip › biosensors-2827077-supplementary.pdf]

Supplementary

Table S1. Kirby Bauer and Optimized Stencil MIC Comparison

Antibiotic	Antibiotic Mass (µg)	A.B. Original Kirby (mm) ± std	A.B Kirby Comparison (mm) ± std	A. B. Stencil (mm) ± std	B. S. Original Kirby (mm) ± std	B. S. Kirby Comparison (mm) ± std	B.S Stencil (mm) ± std	P.A. Original Kirby (mm) ± std	P.A. Kirby Comparison (mm) ± std	P.A Stencil (mm) ± std
Oxytetracycline	7.5	22.38 ± 0.06	7.89 ± 0.06	7.53 ± 0.16	25.73 ± 0.42	9.56 ± 0.42	8.52 ± 0.29	15.19 ± 0.03	4.29 ± 0.03	0.42 ± 0.31
	15	25.57 ± 0.40	9.485 ± 0.40	10.05 ± 0.09	32.34 ± 0.25	12.87 ± 0.25	9.58 ± 0.13	16.59 ± 0.03	4.99 ± 0.03	1.34 ± 0
	30	28.04 ± 0.21	10.72 ± 0.21	11.27 ± 0.3	36.01 ± 0.63	14.71 ± 0.63	10.69 ± 0.21	19.39 ± 0.22	6.39 ± 0.22	4.23 ± 0.66
	60	30.39 ± 0.52	11.89 ± 0.52	12.87 ± 0.08	37.89 ± 0.29	15.64 ± 0.29	11.55 ± 0.12	20.58 ± 0.06	6.99 ± 0.06	6.46 ± 0.29
Kanamycin	7.5	15.44 ± 0.36	4.42 ± 0.36	4.25 ± 0.14	14.2 ± 0.18	3.8 ± 0.18	4.15 ± 0.21	0	0	0
	15	20.11 ± 0.02	6.75 ± 0.02	6.42 ± 0.0.09	17.75 ± 0.26	5.57 ± 0.26	5.39 ± 0.16	8.69 ± 0.47	1.048 ± 0.47	0.42
	30	22.21 ± 0.04	7.81± 0.04	7.89 ± 0.09	20.95 ± 0.23	7.17 ± 0.23	6.61 ± 0.03	11.66 ± 0.43	2.53 ± 0.43	1.78
	60	25.02 ± 0.07	9.21 ± 0.07	9.21 ± 0.21	23.90 ± 0.22	8.65 ± 0.22	7.66 ± 0.13	13.72 ± 0.29	3.56 ± 0.29	3.27
Streptomycin	2.5	0	0	0	0	0	0	8.79 ± 0.38	1.09 ± 0.38	0
	5	0	0	0	0	0	0	13.51 ± 0.08	3.45 ± 0.08	1.93 ± 0.11
	10	0	0	0	0	0	0	17.82 ± 0.68	5.61 ± 0.68	3.75 ± 0.20
	20	1.95 ± 0.48	1.95* ± 0.48	0.64 ± 0.46	7.61 ± 0.10	0.51 ± 0.10	0.85 ± 0.26	21.97 ± 0.48	7.68 ± 0.48	5.27 ± 0.09
Penicillin G.	3	0	0	0		0	0	0	0	0
	6	0	0	0		0	0	0	0	0
	12	0	0	0		0	0	0	0	0
	24	0	0	0	5.13* ± 3.61	5.13* ± 3.61	0.36 ± 0.30	0	0	0

* Not subtracted as stated in [33] as this gives a negative value. A.B. = A. Baumannii, B.S. = B. Subtilis, and P.A. = P. Aeruginosa (calculated with $n = 3$ samples).

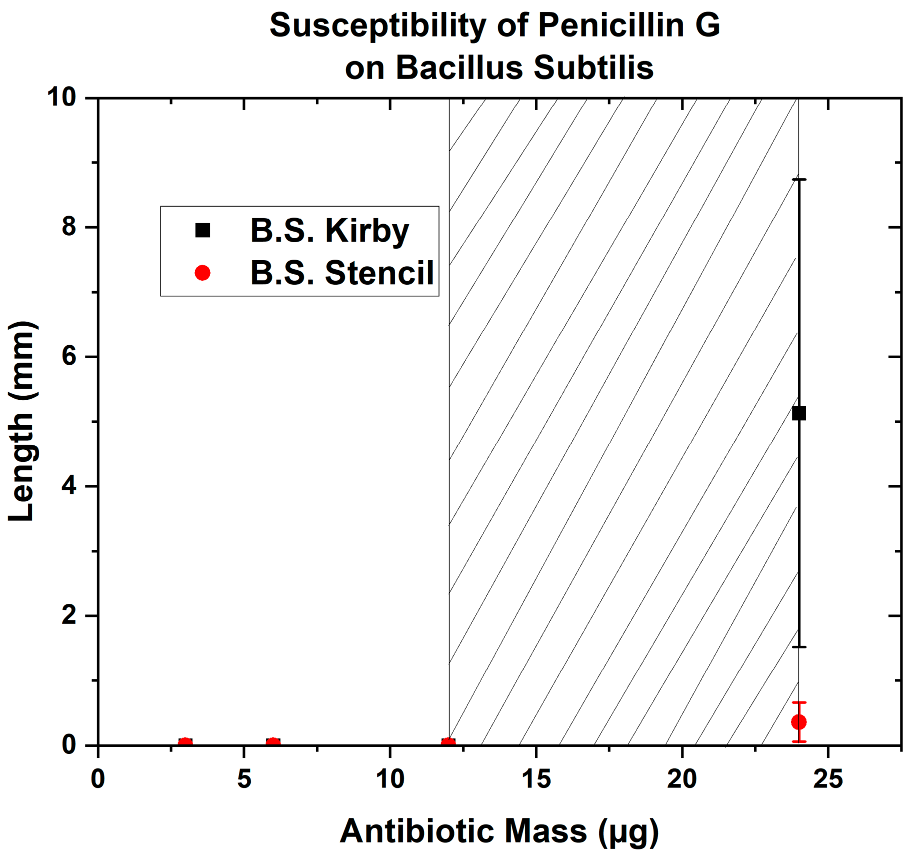

Figure S1. Antibiotic Susceptibility Test of Penicillin G on Bacillus Subtilis.

Penicillin G was found to be unsusceptible to *P. Aeruginosa* and *A. Baumannii* as shown in Table S1.

Table S2. Real Impedance at frequencies 482 Hz, 9.77 kHz and 159.7 kHz for the Control, *A. Baumannii, P. Aeruginosa and B. Subtilis*

Bacteria	Time (Hr)	Real Impedance (482.32 Hz)	STD (±)	Real Impedance (9772.37 Hz)	STD (±)	Real Impedance (159710.45 Hz)	STD (±)
Control	0	276.492	7.226	197.547	7.267	179.751	9.365
	1	244.091	10360	175.608	8.640	158.99	11.266
	2	235.989	11.146	172.495	8.252	156.020	10.617
	4	232.367	14.344	173.269	10.561	157.178	13.005
	6	232.473	8.656	176.526	6.492	160.421	5.610
	8	229.111	7.687	176.526	5.132	161.840	6.384
	12	230.965	9.793	180.258	8.144	165.895	9.810
	18	229.276	10.975	179.508	9.749	164.606	11.053
	24	232.065	11.698	182.658	10.202	167.294	10.345
	36	239.591	11.446	188.747	9.058	172.869	9.399
	48	259.530	34.563	205.435	30.585	187.218	30.805
Acinetobacter Baumannii (OD_{600} = 0.1)	0	292.844	41.682	209.861	37.773	188.225	33.975
	1	275.850	25.96	200.662	26.769	181.868	24.223
	2	268.194	20.565	197.756	23.836	180.052	22.346
	4	265.249	17.052	197.706	21.677	179.969	20.597
	6	279.037	28.116	207.273	23.0315	189.638	22.537
	8	256.904	14.997	198.329	18.928	181.361	19.130
	12	266.330	14.532	209.009	18.170	189.960	19.413
	18	265.574	12.992	211.102	16.277	192.769	17.686
	24	277.985	4.563	222.079	7.106	202.152	10.881
	36	307.529	5.840	246.752	2.15	222.828	7.040
	48	1165.271	967.286	1013.146	858.396	959.730	828.594
Pseudomonas Aeruginosa (OD_{600} = 0.4)	0	299.506	12.751	225.497	1.010	206.158	6.989
	1	268.832	22.943	207.278	17.747	190.750	19.901
	2	240.781	29.010	186.727	17.564	171.571	14.787
	4	223.316	26.404	175.363	18.008	162.272	15.569

	6	213.857	26.0477	169.869	19.250	158.139	17.049
	8	204.077	16.342	164.039	13.504	153.825	13.250
	12	214.685	10.866	175.770	10.009	165.131	10.947
	18	324.526	172.936	282.433	165.055	270.621	162.482
	24	956.0166	1056.186	811.797	904.729	763.694	851.870
	36	22348.171	6754.372	14397.180	2377.514	11628.994	653.949
	48	40138.749	15990.509	24911.153	6884.693	16795.947	1455.167
Bacillus Subtilis (OD_{600} = 1.0)	0	347.365	57.098	260.747	45.198	235.88995	41.224
	1	264.621	48.641	203.323	37.726	185.699	34.472
	2	253.146	54.214	200.816	44.585	186.315	41.890
	4	235.188	42.450	192.216	34.126	179.959	31.467
	6	256.045	39.727	213.925	33.317	201.070	31.153
	8	214.871	37.504	181.610	32.598	171.503	30.773
	12	207.288	35.699	175.304	31.260	165.325	29.377
	18	204.899	33.989	173.220	29.492	162.755	27.366
	24	209.906	30.790	177.177	26.628	165.444	25.046
	36	206.257	25.692	173.922	22.402	161.745	20.895
	48	204.379	24.074	172.246	21.458	159.535	20.382

Table S3. Imaginary Impedance at frequencies 482.32 Hz, 9.77 kHz and 159.7 kHz for the Control, *A. Baumannii, P. Aeruginosa and B. Subtilis*

Bacteria	Time (Hr)	Imaginary Impedance (482.32 Hz)	STD (±)	Imaginary Impedance (9772.37 Hz)	STD (±)	Imaginary Impedance (159710.45 Hz)	STD (±)
Control	0	-146.338	14.774	-41.812	2.261	-8.254	2.391
	1	-128.463	11.853	-37.268	1.899	-6.839	1.585
	2	-120.594	8.085	-35.119	1.144	-6.570	1.300
	4	-115.868	5.871	-33.173	1.157	-6.521	1.309
	6	-112.974	5.968	-31.793	1.661	-6.629	1.553
	8	-109.194	6.209	-29.726	2.159	-6.443	1.474
	12	-107.818	6.298	-28.590	2.237	6.634	1.689
	18	-108.202	5.955	-28.438	2.262	-7.157	1.935
	24	-109.778	6.768	-28.603	2.821	-7.613	2.409
	36	-113.316	8.260	-29.346	3.227	-8.039	2.542
	48	-121.260	13.39	-31.793	3.634	-9.426	2.494
Acinetobacter Baumannii (OD_{600} = 0.1)	0	-159.391	2.055	-46.157	1.841	-9.5135	1.326
	1	-147.657	2.506	-41.465	0.558	-8.113	0.732
	2	-137.349	2.395	-38.329	0.788	-7.871	0.463
	4	-128.552	3.677	-36.596	1.109	-8.161	0.598
	6	-128.014	2.361	-35.235	1.821	-8.358	0.439
	8	-110.976	4.423	-32.155	1.356	-8.119	0.302
	12	-112.162	4.801	-32.381	1.756	-9.423	0.290
	18	-111.460	5.358	-30.898	1.936	-9.241	0.781
	24	-118.548	7.993	-32.073	3.824	-10.298	1.312
	36	-131.118	7.182	-35.630	5.338	-12.512	2.821
	48	-270.773	168.836	-73.755	42.497	-31.550	20.523
Pseudomonas Aeruginosa (OD_{600} = 0.4)	0	-137.851	16.818	-40.978	7.389	-7.959	2.629
	1	-124.482	17.597	-35.520	7.483	-6.888	2.506
	2	-115.789	16.958	-31.510	7.184	-6.334	2.447

	4	-109.681	13.795	-28.054	6.089	-5.785	2.229
	6	-104.720	13.639	-25.587	5.912	-5.571	2.280
	8	-98.179	8.139	-23.005	4.006	-5.257	1.735
	12	-97.319	8.142	-22.644	3.903	-5.713	1.816
	18	-115.240	31.612	-24.718	6.003	-6.924	2.731
	24	-236.469	200.619	-68.169	65.991	-28.927	32.652
	36	-11409.902	5539.428	-3512.750	1719.755	-1735.417	976.137
	48	-20443.405	10538.206	-8324.228	4785.135	-4459.828	2506.907
Bacillus Subtilis (OD_{600} = 1.0)	0	-154.467	22.531	-47.868	7.267	-10.076	1.788
	1	-120.047	17.086	-34.742	5.786	-6.767	1.497
	2	-111.207	16.287	-29.966	5.615	-6.078	1.437
	4	-96.508	14.317	-24.345	4.585	-5.559	1.236
	6	-93.232	12.188	-23.391	3.547	-6.137	1.193
	8	-74.948	8.561	-18.114	2.511	-5.075	1.116
	12	-70.612	7.537	-17.607	2.563	-5.040	1.066
	18	-68.197	7.427	-17.438	2.433	-5.257	1.151
	24	-68.928	6.938	-18.145	2.180	-6.030	0.816
	36	-66.529	4.678	-17.920	1.704	-6.240	0.693
	48	-62.717	3.159	-17.528	1.090	-6.533	0.643

Table S4. Phase at frequencies 482 Hz, 9.77 kHz and 159.7 kHz for the Control, *A. Baumannii, P. Aeruginosa and B. Subtilis*

Bacteria	Time (Hr)	Phase (482.32 Hz)	STD (±)	Phase (9772.37 Hz)	STD (±)	Phase (159710.45 Hz)	STD (±)
Control	0	-27.818	1.479	-11.950	0.478	-2.663	0.885
	1	-27.718	2.786	-12.002	0.739	-2.516	0.781
	2	-27.054	2.757	-11.523	0.465	-2.452	0.654
	4	-26.541	2.802	-10.871	0.656	-2.434	0.715
	6	-25.903	2.865	-10.218	0.161	-2.365	0.543
	8	-25.467	3.016	-9.552	0.553	-2.298	0.603
	12	-25.019	3.209	-9.019	0.707	-2.323	0.709
	18	-25.269	3.467	-9.019	0.770	-2.521	0.763
	24	-25.308	3.793	-8.901	0.759	-2.627	0892
	36	-25.283	4.252	-8.827	0.788	-2.684	0.912
	48	-25.107	4.909	-8.871	0.853	-2.953	0.921
Acinetobacter Baumannii (OD_{600} = 0.1)	0	-28.747	3.788	-12.695	1.724	-2.917	0.176
	1	-27.979	2.949	- 11.858	1.455	-2.569	0.136
	2	-27.002	2.384	-11.136	1.465	-2.524	0.182
	4	-26.113	1.751	-10.626	1.358	-2.615	0.219
	6	-24.426	1.968	-9.787	1.371	-2.547	0.207
	8	-24.053	1.306	-9.321	1.227	-2.581	0.170
	12	-23.539	1.299	-8.907	1.221	-2.868	0.291
	18	-13.251	1.477	-8.412	1.150	-2.769	0.353
	24	-23.117	1.264	-8.248	1.187	-2.943	0.511
	36	-22.635	1.175	-8.218	1.256	-3.237	0.802
	48	-16.627	4.261	-5.547	2.182	-2.514	1.174
Pseudomonas Aeruginosa (OD_{600} = 0.4)	0	-24.615	1.775	-10.296	1.862	-2.236	0.790
	1	-24.802	2.395	-9.794	2.119	-2.106	0.778
	2	-25.639	1.694	-9.530	1.678	-2.094	0.698

	4	-26.178	1.768	-9.075	1.568	-2.028	0.675
	6	-26.112	1.641	-8.562	1.571	-2.009	0.722
	8	-25.726	1.636	-8.027	1.423	-1.975	0.644
	12	-24.369	1.514	-7.370	1.422	-2.009	0.722
	18	-21.700	4.475	-6.062	2.220	-1.745	0.842
	24	-20.215	5.847	-6.250	1.712	-2.152	0.789
	36	-25.693	4.102	-12.989	3.93	-8.212	4.088
	48	-25.901	2.905	-17.066	4.697	-14.131	6.353
Bacillus Subtilis (OD_{600} = 1.0)	0	-24.040	0.675	-10.451	0.538	-2.448	0.114
	1	-24.548	0.834	-9.728	0.190	-2.076	0.096
	2	-23.982	1.324	-8.548	0.335	-1.866	0.084
	4	-22.420	0.719	-7.207	0.124	-1.757	0.098
	6	-20.086	0.517	-6.244	0.029	-1.736	0.079
	8	-19.395	0.995	-5.734	0.215	-1.684	0.062
	12	-18.987	1.103	-5.766	0.179	-1.736	0.055
	18	-18.553	0.901	-5.776	0.172	-1.835	0.088
	24	-18.282	0.726	-5.872	0.166	-2.094	0.121
	36	-17.979	0.855	-5.908	0.222	-2.214	0.049
	48	-17.190	1.229	-5.855	0.362	-2.358	0.165

Table S5. Capacitance at frequencies 482 Hz, 9.77 kHz and 159.7 kHz for the Control, *A. Baumannii, P. Aeruginosa and B. Subtilis*

Bacteria	Time (Hr)	Capacitance (F) (@ 482.32 Hz)	STD (F)	Capacitance (F) (@ 9772.37 Hz)	STD (F)	Capacitance (F) (@ 159710.45 Hz)	STD
Control	0	2.278×10^{-6}	2.322×10^{-7}	3.906×10^{-7}	2.162×10^{-8}	1.298×10^{-7}	3.135×10^{-8}
	1	2.589×10^{-6}	2.262×10^{-7}	4.381×10^{-7}	2.231×10^{-8}	1.529×10^{-7}	3.123×10^{-8}
	2	2.748×10^{-6}	1.766×10^{-7}	4.642×10^{-7}	1.507×10^{-8}	1.570×10^{-7}	2.737×10^{-8}
	4	2.855×10^{-6}	1.407×10^{-7}	4.915×10^{-7}	1.696×10^{-8}	1.584×10^{-7}	2.794×10^{-8}
	6	2.929×10^{-6}	1.596×10^{-7}	5.137×10^{-7}	2.785×10^{-8}	1.577×10^{-7}	3.181×10^{-8}
	8	3.032×10^{-6}	1.775×10^{-7}	5.509×10^{-7}	4.183×10^{-8}	1.620×10^{-7}	3.245×10^{-8}
	12	3.071×10^{-6}	1.871×10^{-7}	5.733×10^{-7}	4.730×10^{-8}	1.589×10^{-7}	3.445×10^{-8}
	18	3.058×10^{-6}	1.683×10^{-7}	5.761×10^{-7}	4.395×10^{-8}	1.485×10^{-7}	3.465×10^{-8}
	24	3.017×10^{-6}	1.914×10^{-7}	5.748×10^{-7}	5.600×10^{-8}	1.427×10^{-7}	3.756×10^{-8}
	36	2.928×10^{-6}	2.244×10^{-7}	5.618×10^{-7}	6.252×10^{-8}	1.351×10^{-7}	3.541×10^{-8}
	48	2.755×10^{-6}	3.094×10^{-7}	5.196×10^{-7}	6.443×10^{-8}	1.128×10^{-7}	2.721×10^{-8}
Acinetobacter Baumannii ($OD_{600} = 0.1$)	0	2.089×10^{-6}	4.601×10^{-8}	3.533×10^{-7}	1.376×10^{-8}	1.066×10^{-7}	1.359×10^{-8}
	1	2.273×10^{-6}	7.898×10^{-8}	3.928×10^{-7}	5.341×10^{-9}	1.237×10^{-7}	1.050×10^{-8}
	2	2.43×10^{-6}	6.890×10^{-8}	4.250×10^{-7}	8.655×10^{-9}	1.270×10^{-7}	7.183×10^{-8}
	4	2.548×10^{-6}	6.277×10^{-8}	4.454×10^{-7}	1.322×10^{-8}	1.227×10^{-7}	8.883×10^{-8}
	6	2.624×10^{-6}	5.874×10^{-7}	4.634×10^{-7}	2.459×10^{-8}	1.195×10^{-7}	6.385×10^{-8}
	8	2.888×10^{-6}	1.213×10^{-7}	5.073×10^{-7}	2.133×10^{-8}	1.229×10^{-7}	4.566×10^{-9}
	12	2.854×10^{-6}	1.269×10^{-7}	5.044×10^{-7}	2.707×10^{-8}	1.058×10^{-7}	3.192×10^{-9}
	18	2.902×10^{-6}	1.215×10^{-7}	5.291×10^{-7}	3.333×10^{-8}	1.085×10^{-7}	8.659×10^{-9}
	24	2.790×10^{-6}	1.762×10^{-7}	5.148×10^{-7}	5.952×10^{-8}	9.851×10^{-8}	1.371×10^{-8}
	36	2.583×10^{-6}	1.901×10^{-7}	4.678×10^{-7}	7.228×10^{-8}	8.476×10^{-8}	2.272×10^{-8}
	48	1.565×10^{-6}	6.613×10^{-7}	2.893×10^{-7}	1.192×10^{-7}	4.500×10^{-8}	2.108×10^{-8}
	0	2.431×10^{-6}	3.128×10^{-7}	4.124×10^{-7}	8.369×10^{-8}	1.438×10^{-7}	5.731×10^{-8}

Pseudomonas Aeruginosa (OD_{600} = 0.4)	1	2.702×10^{-6}	3.696×10^{-7}	4.796×10^{-7}	1.014×10^{-7}	1.651×10^{-7}	5.822×10^{-8}
	2	2.911×10^{-6}	4.202×10^{-7}	5.447×10^{-7}	1.240×10^{-7}	1.829×10^{-7}	6.878×10^{-8}
	4	3.054×10^{-6}	3.700×10^{-7}	6.075×10^{-7}	1.258×10^{-7}	1.980×10^{-7}	6.904×10^{-8}
	6	3.200×10^{-6}	3.834×10^{-7}	6.683×10^{-7}	1.385×10^{-7}	2.076×10^{-7}	7.184×10^{-8}
	8	3.383×10^{-6}	2.724×10^{-7}	7.297×10^{-7}	1.260×10^{-7}	2.112×10^{-7}	6.766×10^{-8}
	12	3.415×10^{-6}	2.977×10^{-7}	7.430×10^{-7}	1.390×10^{-7}	1.980×10^{-7}	7.539×10^{-8}
	18	3.061×10^{-6}	7.242×10^{-7}	7.016×10^{-7}	1.786×10^{-7}	1.765×10^{-7}	8.503×10^{-8}
	24	2.540×10^{-6}	1.353×10^{-6}	5.521×10^{-7}	3.365×10^{-7}	1.402×10^{-7}	1.127×10^{-7}
	36	3.572×10^{-8}	1.450×10^{-8}	5.670×10^{-7}	2.136×10^{-9}	7.547×10^{-10}	3.310×10^{-10}
	48	2.039×10^{-8}	8.533×10^{-9}	2.588×10^{-9}	1.126×10^{-9}	2.942×10^{-10}	1.304×10^{-10}
Bacillus Subtilis (OD_{600} = 1.0)	0	2.178×10^{-6}	2.884×10^{-7}	3.474×10^{-7}	4.763×10^{-8}	1.017×10^{-7}	1.606×10^{-8}
	1	2.800×10^{-6}	3.648×10^{-7}	4.807×10^{-7}	7.199×10^{-8}	1.537×10^{-7}	2.942×10^{-8}
	2	3.026×10^{-6}	4.027×10^{-7}	5.608×10^{-7}	9.282×10^{-8}	1.722×10^{-7}	3.495×10^{-8}
	4	3.488×10^{-6}	4.702×10^{-7}	6.907×10^{-7}	1.159×10^{-7}	1.872×10^{-7}	3.601×10^{-8}
	6	3.607×10^{-6}	5.196×10^{-7}	7.146×10^{-7}	1.213×10^{-7}	1.698×10^{-7}	3.827×10^{-8}
	8	4.456×10^{-6}	4.714×10^{-7}	9.151×10^{-7}	1.164×10^{-7}	2.049×10^{-7}	3.911×10^{-8}
	12	4.723×10^{-6}	4.692×10^{-7}	9.430×10^{-7}	1.245×10^{-7}	2.057×10^{-7}	3.790×10^{-8}
	18	4.892×10^{-6}	4.949×10^{-7}	9.508×10^{-7}	1.214×10^{-7}	1.977×10^{-7}	3.766×10^{-8}
	24	4.833×10^{-6}	4.556×10^{-7}	9.096×10^{-7}	1.008×10^{-7}	1.682×10^{-7}	2.254×10^{-8}
	36	4.983×10^{-6}	3.368×10^{-7}	9.165×10^{-7}	8.170×10^{-8}	1.616×10^{-7}	1.721×10^{-8}
	48	5.275×10^{-6}	2.755×10^{-7}	9.327×10^{-7}	5.878×10^{-8}	1.541×10^{-7}	1.631×10^{-7}

Below are timepoints and images from the confocal and SEM microscope with the appropriate real, imaginary, phase angle and capacitive values.

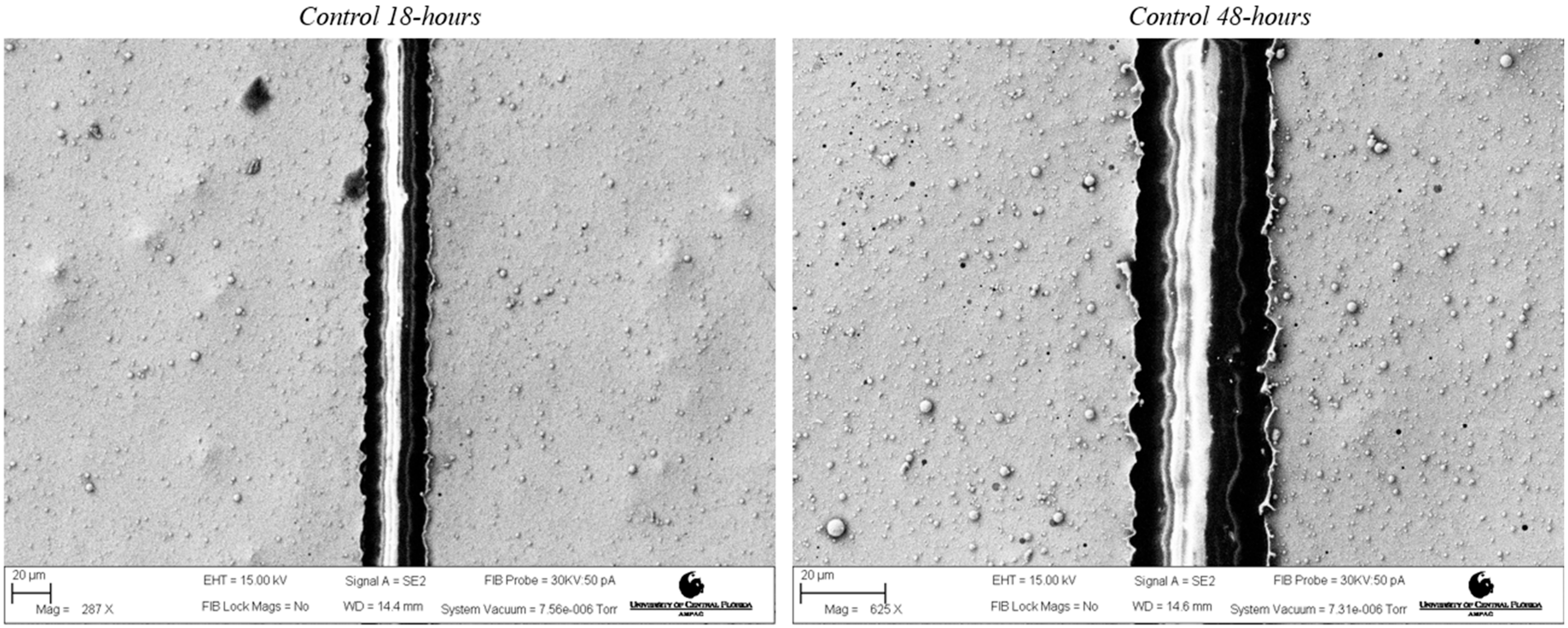

Figure S2. (A) SEM image of the control IDE after 18 hours. (B) Confocal Height map of the control IDE after 48-hours, showing no change on the surface of the IDE or in the spacing.

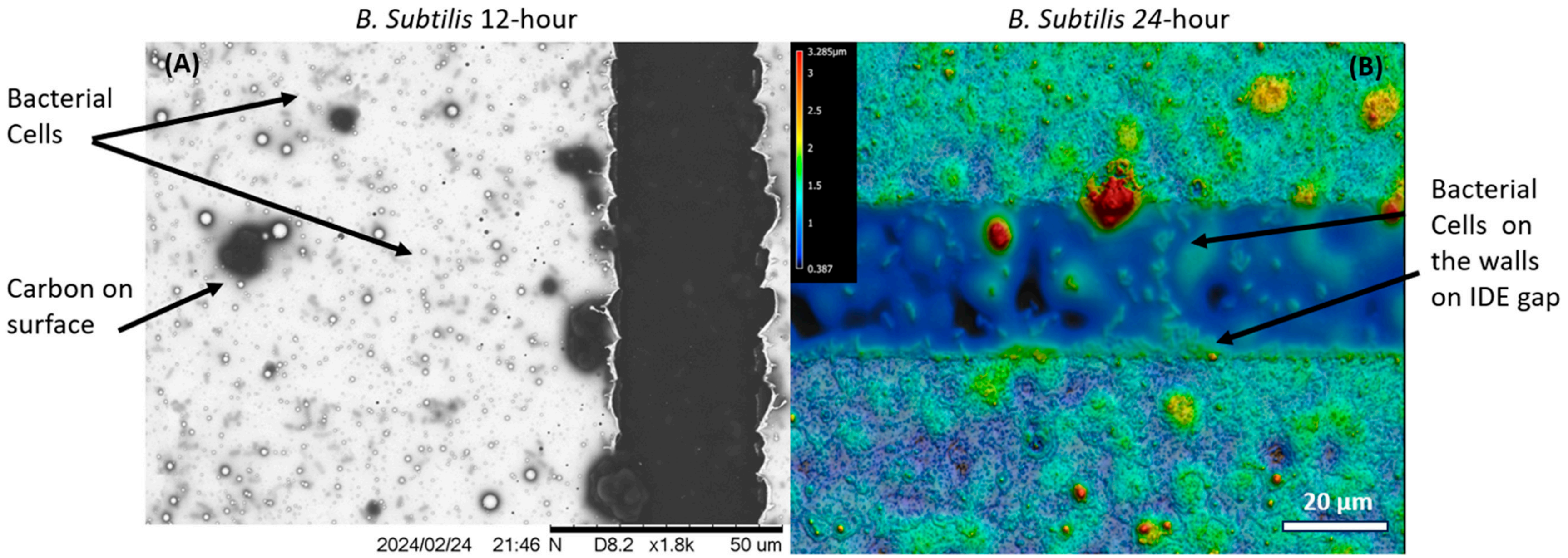

Figure S3. (A) SEM image of B. Subtilis 12-hour bacterial growth. (B) Confocal Height map of B. Subtilis 24-hour, showing cell growth in IDE gap.

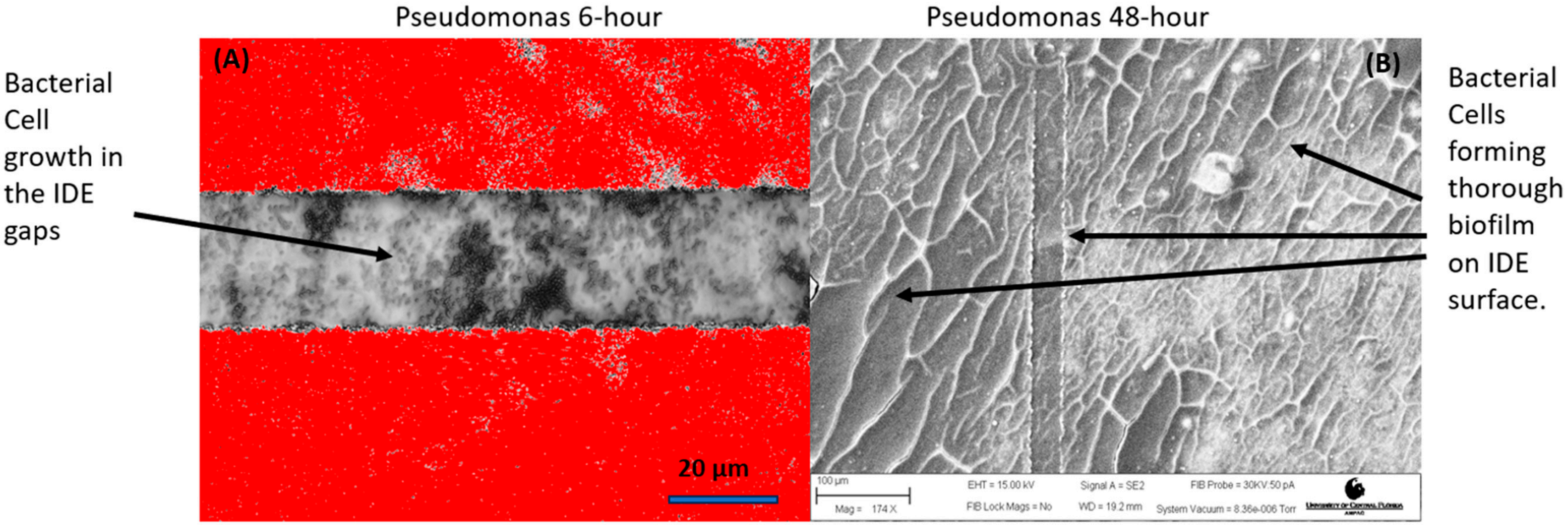

Figure S4. (A) Laser Confocal Image showing biofilm growth in the IDE gap. (B) SEM picture showing IDE surface covered with biofilm.

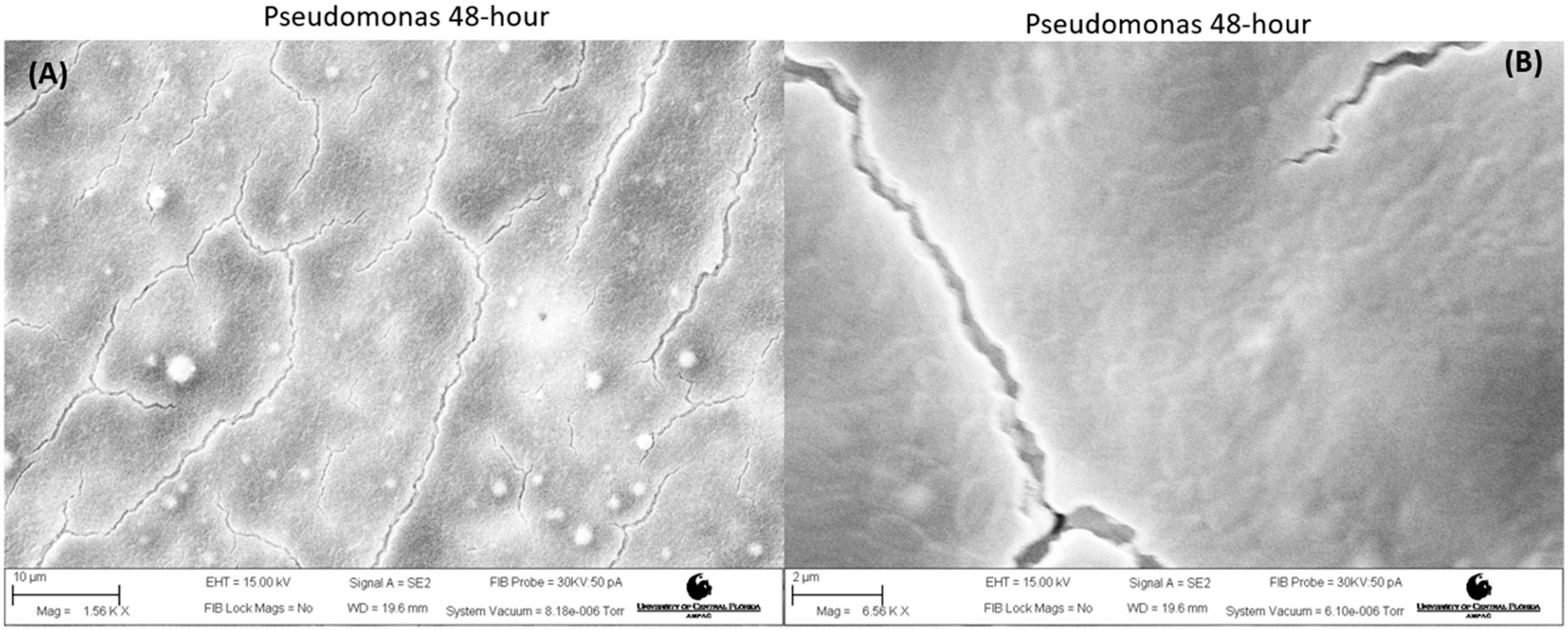

Figure S5. (A) SEM close up of Pseudomonas Aeruginosa dried biofilm growth. (B) Close up of dried biofilm growth.

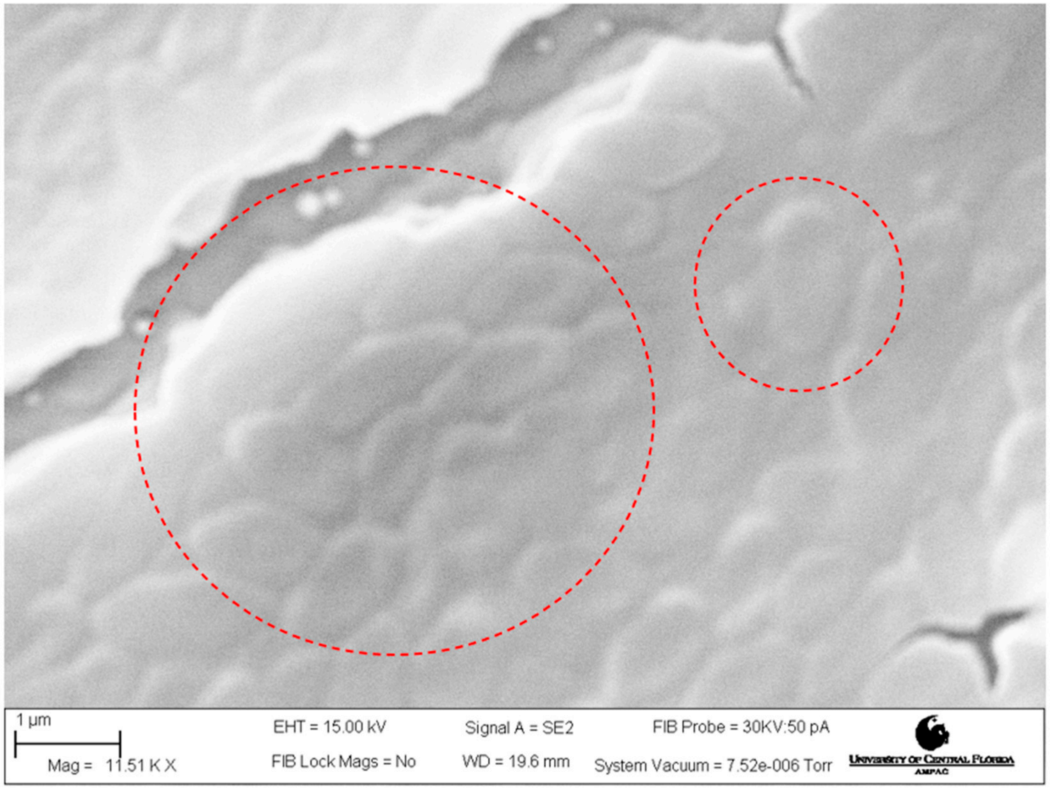

Figure S6. SEM close up biofilm growth showing individual cells forming biofilm for Pseudomonas Aeruginosa.

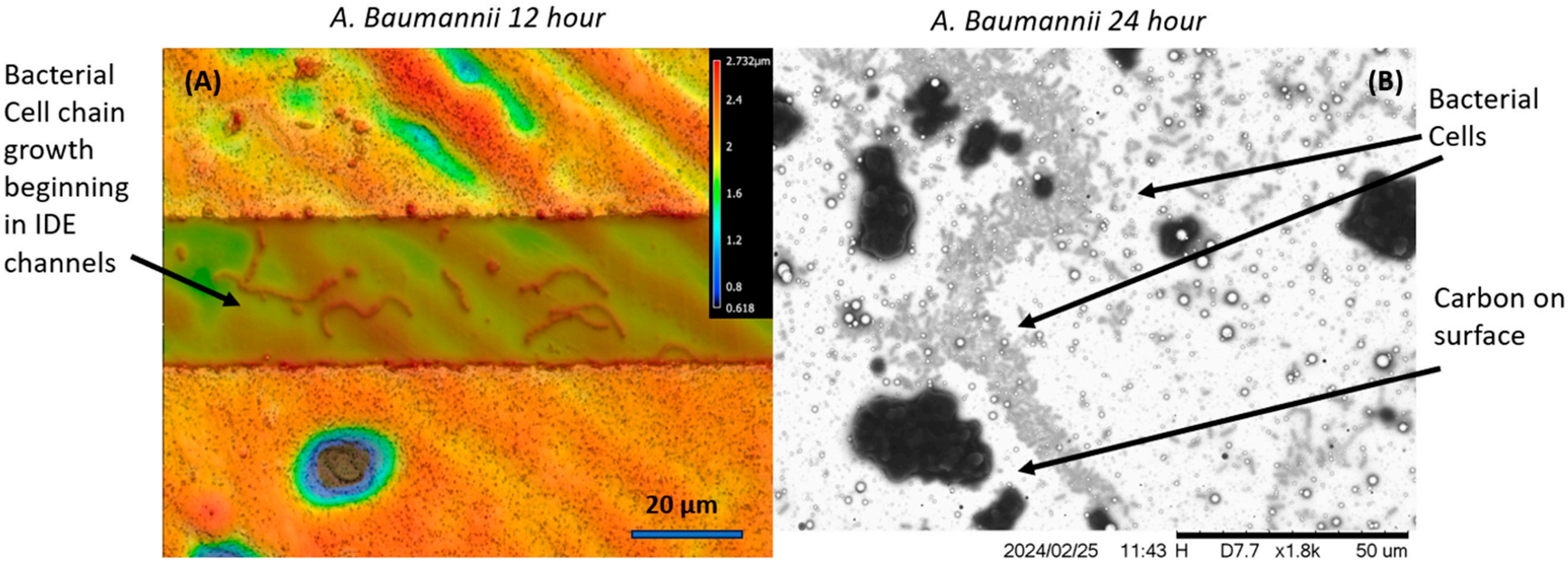

Figure S7. (A) Confocal Height Image of the interdigitated electrode gap with A. Baumannii cell chains forming. (B) SEM image showing A. Baumannii biofilm cells at the 24-hour point.

Searching the Query

In order to view the database inputs and determine whether our theory bacterial databases development would have any use, the results were also queried against the MIC stencil antibiotics and the corresponding pharmaceuticals would be useful. The resulting search query from the above IDE sharded example leads to a dropdown menu for the user as shown in figure S7.

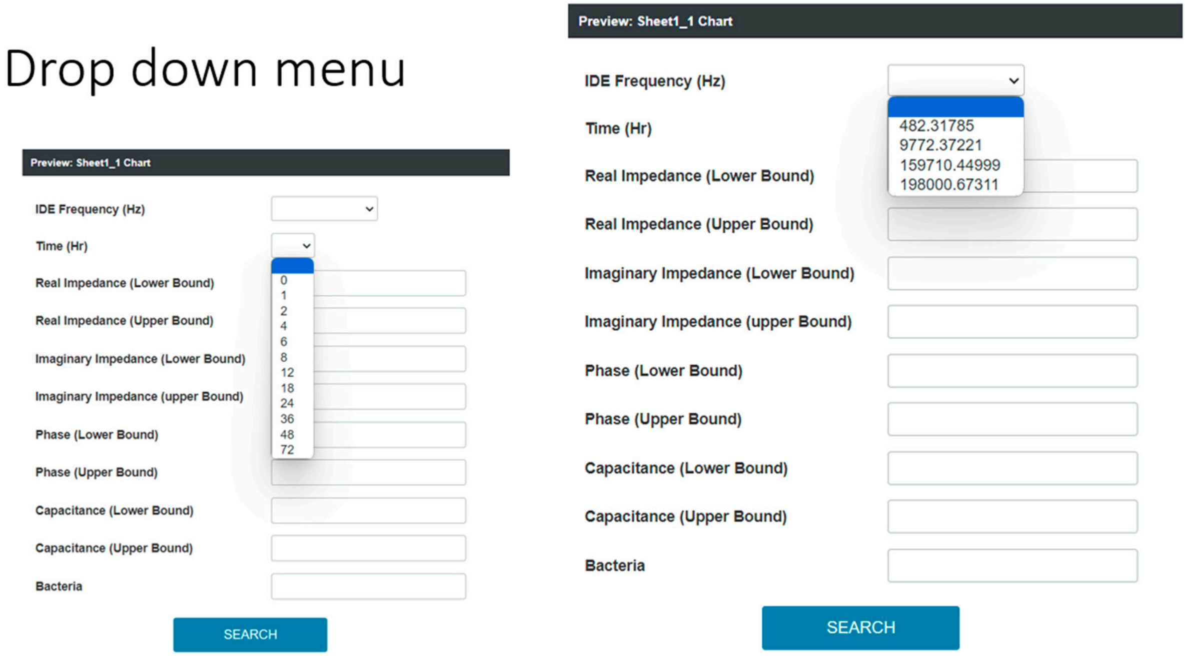

FigureS8. Interdigitated electrode partitioned database drop down and enterable standard deviation search menu.

IDE Frequency (Hz) 9772.37221

Time (Hr) 8

Real Impedance (Lower Bound)

Real Impedance (Upper Bound)

Imaginary Impedance (Lower Bound)

Imaginary Impedance (upper Bound)

Phase (Lower Bound)

Phase (Upper Bound)

Capacitance (Lower Bound)

Capacitance (Upper Bound)

Bacteria

SEARCH

IDE Frequency (Hz)	Time (Hr)	Real Impedance (Ω)	STD	Imaginary Impedance (Ω)	STD	Phase (°)	STD1	Capacitance (F)	STD2	Bacteria	
9772.37221	8	180.53005	33.10215	-18.45695	33.10215	-5.8819	0.246	0.000001	0	Bacillus Subtillis	View Details
9772.37221	8	161.49612	16.12494	-23.7872	16.12494	-8.42119	1.52781	0.000001	0	Psuedomonas Aeruginosa	View Details
9772.37221	8	198.32984	18.92869	-32.15516	18.92869	-9.32108	1.22754	0.000001	0	Acinetobacter Baumannii	View Details

Records 1-3 of 3

Figure S9. Using the dropdown menu for the Interdigitated electrode sharded database drop down menu.

159710.44999	0	237.92333	41.86321	-10.19948	41.86321	-2.51573	0.1927	0	0	Bacillus Subtillis	View Details
159710.44999	1	187.48576	37.81173	-7.11385	37.81173	-2.16002	0.10506	0	0	Bacillus Subtillis	View Details
159710.44999	36	160.39131	22.15945	-5.94686	22.15945	-2.12858	0.07431	0	0	Bacillus Subtillis	View Details
159710.44999	48	156.60463	22.02985	-6.163	22.02985	-2.26621	0.11201	0	0	Bacillus Subtillis	View Details
159710.44999	72	187.11446	50.4265	-7.62546	50.4265	-2.28468	0.13532	0	0	Bacillus Subtillis	View Details
198000.67311	0	237.24561	41.75944	-9.32492	41.75944	-2.30712	0.18486	0	0	Bacillus Subtillis	View Details
198000.67311	36	159.79047	22.1174	-5.57127	22.1174	-2.00145	0.08734	0	0	Bacillus Subtillis	View Details
198000.67311	48	155.94624	21.99444	-5.80146	21.99444	-2.14103	0.10589	0	0	Bacillus Subtillis	View Details
198000.67311	72	186.22578	50.15264	-7.37911	50.15264	-2.216	0.14795	0	0	Bacillus Subtillis	View Details
9772.37221	2	184.99193	20.22294	-32.70757	20.22294	-9.98105	1.69927	0.000001	0	Psuedomonas Aeruginosa	View Details
9772.37221	4	174.03885	20.11954	-29.05042	20.11954	-9.45678	1.62863	0.000001	0	Psuedomonas Aeruginosa	View Details
9772.37221	6	167.79159	20.35586	-26.27327	20.35586	-8.90082	1.60332	0.000001	0	Psuedomonas Aeruginosa	View Details
9772.37221	8	161.49612	16.12494	-23.7872	16.12494	-8.42119	1.52781	0.000001	0	Psuedomonas Aeruginosa	View Details
9772.37221	12	148.0709	27.40616	-22.70951	27.40616	-7.87598	1.41096	0.000001	0	Psuedomonas Aeruginosa	View Details
9772.37221	18	336.44741	246.6231	-33.66994	246.6231	-6.09707	2.53068	0.000001	0	Psuedomonas Aeruginosa	View Details
9772.37221	24	961.31271	1108.9165	-150.24545	1108.9165	-6.79283	1.45751	0.000001	0	Psuedomonas Aeruginosa	View Details
159710.44999	0	209.3616	5.55301	-8.36711	5.55301	-2.31143	0.82344	0	0	Psuedomonas Aeruginosa	View Details
159710.44999	1	189.11385	20.75583	-7.08943	20.75583	-2.18646	0.80592	0	0	Psuedomonas Aeruginosa	View Details
159710.44999	2	169.09002	17.24457	-6.4879	17.24457	-2.17506	0.71958	0	0	Psuedomonas Aeruginosa	View Details

Figure S10. Not using the dropdown menu and including all hours leads to a multitude of options for bacterial discovery.

In **Figure S9** it is observed how using the dropdown menu leads to a limited number of results, depicting the three studied bacteria at the 9.77 kHz frequency point, while also revealing the real impedance, imaginary impedance, capacitance and phase at the selected 8-hour mark. If a time or a frequency point was not selected, every entry point in the database would be shown with the option to scroll the system **Figure S10**. This same instance is shown when using the sharded susceptibility database in **Figure S11**. In this instance an antibiotic mass of 7.5 µg was selected, leading to a table that includes every antibiotic that uses a mass of 7.5 µg which in BacteSign are Oxytetracycline and Kanamycin. In this instance a public health provider could scroll the database and make a decision with his/her resultant susceptibility test themselves, or they could take the

next step and manually enter the lower and upper bound values with their calculated standard deviation shown in **Figure S12**. Entering a bound for the sample under test, improves the search criteria where for an example usage of 6 mm and 10 mm values for an upper and lower bound for the search, leading to a result only two of the three possible bacterial pathogens would be the result. If the search criteria were made tighter such as 8 mm and 10 mm it leads to one solution of bacteria, in this case being B. Subtilis.

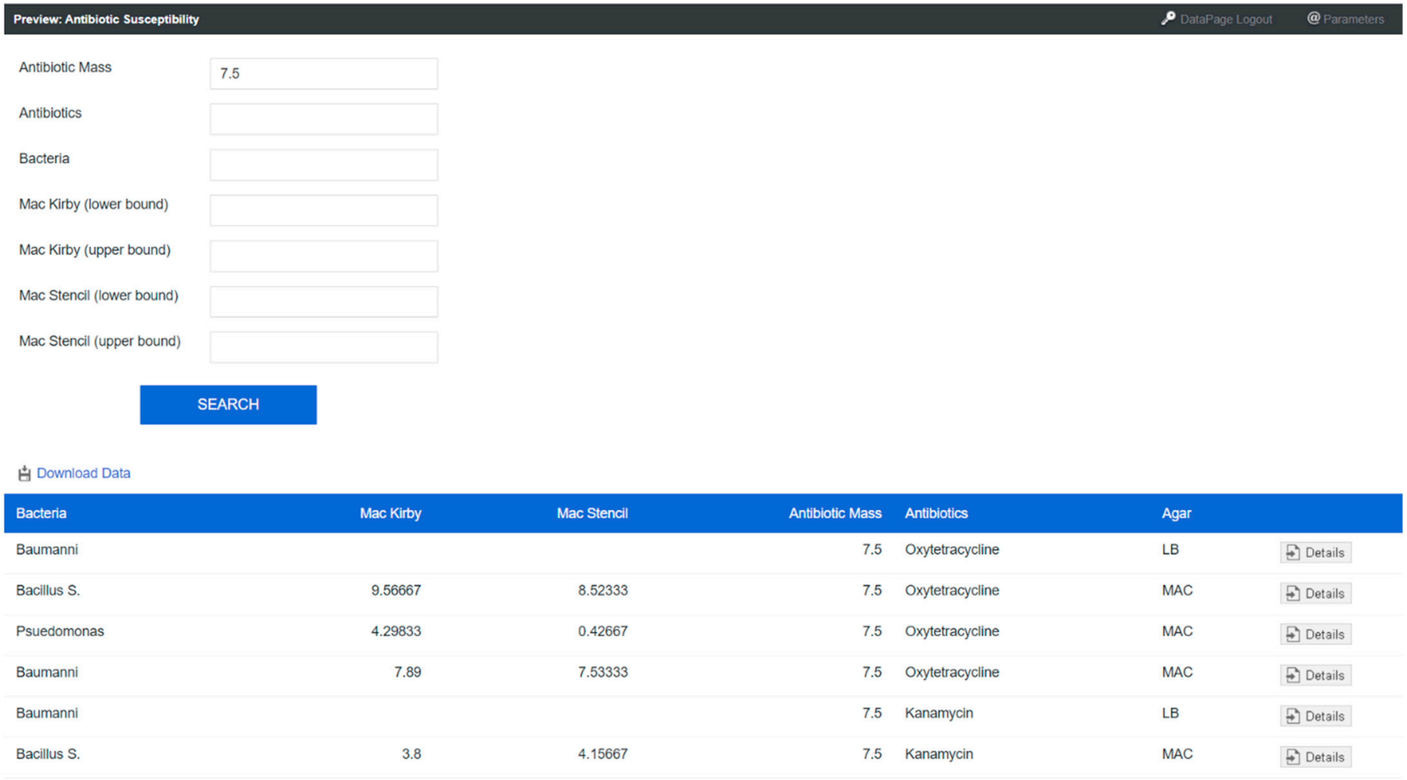

Figure S11: Entering an Antibiotic Mass and limiting the search with a lower and upper bound Susceptibility test values.

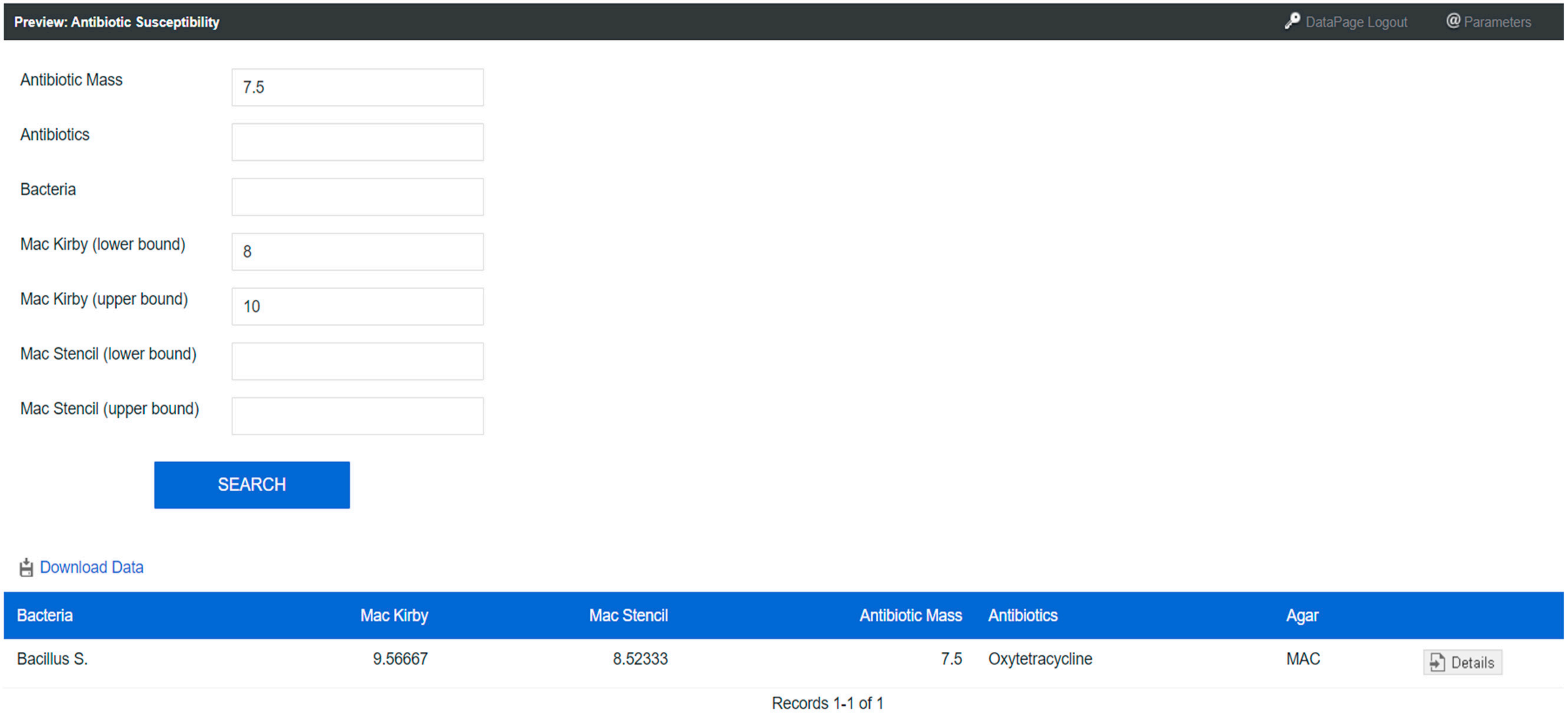

Figure S12: Using lower and upper bounds as a manual entrance for standard deviation.
